# Regulation of NK cell development, maturation, and antitumor responses by the nuclear receptor NR2F6

**DOI:** 10.1038/s41419-025-07407-4

**Published:** 2025-02-07

**Authors:** Johannes Woelk, Florian Hornsteiner, Stephanie Aschauer-Wallner, Patrizia Stoitzner, Gottfried Baier, Natascha Hermann-Kleiter

**Affiliations:** 1 Institute of Cell Genetics, Department for Genetics and Pharmacology, Medical University of Innsbruck, Innsbruck, Austria; 2 Department of Dermatology, Venereology & Allergology, Medical University of Innsbruck, Innsbruck, Austria; 3 Laboratory of Tumor Immunology, Tyrolean Cancer Institute & Internal Medicine V, Medical University of Innsbruck, 6020 Innsbruck, Austria; 4 Present Address: Regenerative Medicine, Spinal Cord Injury and Tissue Regeneration Center Salzburg, Paracelsus Medical University Salzburg, Salzburg, Austria

**Keywords:** Immunosurveillance, Innate lymphoid cells

## Abstract

Natural killer (NK) cell development and functionality rely on precise regulation by specific transcription factors (TFs). Our study demonstrates that the nuclear orphan receptor NR2F6 represses the expression of the activating receptor NKp46, an established key player in NK cell-mediated cytotoxicity during infection and tumor rejection. Despite normal NK cell development in the bone marrow, germline *Nr2f6*-deficient mice exhibit impaired terminal maturation of NK cells in the periphery. Short-term NK cell responses to lipopolysaccharide (LPS) activation, independent of NKp46, are subsequently reduced in *Nr2f6*-deficient mice. Conventional type 1 dendritic cells (cDC1) and macrophage populations are decreased in spleens of *Nr2f6*-deficient mice, subsequently, IL-15-dependent NK cell priming is limited. Administration of exogenous IL-15 in vitro and as IL-15 complex in vivo can compensate for these deficits, promoting terminal maturation of NK cells in *Nr2f6*-deficient mice. Subsequent transcriptome analysis reveals significant changes in gene expression profiles of NK cells from IL-15 complex treated *Nr2f6*-deficient mice, with notable alterations in essential NK genes such as *Klrg1, Prdm1, Stat5a, Zeb2*, and *Prf1*. Consequently, *Nr2f6*-deficient IL-15 complex-treated NK cells raise enhanced effector responses of IFNγ, Perforin, and Granzyme B upon ex vivo activation. Of importance, *Nr2f6*-deficient mice are protected against MHC-I negative B16-F10 melanoma lung metastasis formation, especially with IL-15 complex treatment, indicating the potential of NR2F6 to affect NKp46-dependent NK cell-mediated tumor surveillance. The therapeutic targeting of NR2F6 may be a promising strategy for boosting NKp46-dependent NK-cell-mediated tumor surveillance and metastasis.

## Introduction

Natural killer (NK) cells are pivotal in regulating microbial infections, tumor surveillance, allograft rejection, pregnancy, and autoimmune responses [1–3]. Their inherent antitumor activity, independent of neo-antigen exposure, makes them crucial in preventing metastasis and a prime target in immuno-oncology therapies [4–7]. NK cell function is modulated by a diverse array of germline-encoded surface receptors, either activating or inhibiting, facilitating discrimination between “self” and “non-self” [8]. NK cells detect aberrantly expressed stress-induced ligands on unhealthy or major histocompatibility complex (MHC) class I-deficient cells [7, 9, 10].

The natural cytotoxicity receptors (NCRs) were the first identified for their ability to induce NK cell cytotoxicity against tumor cells. Functionally, genetic deficiency of the NCR member NKp46, encoded by *Ncr1*, leads to impaired clearance of subcutaneous melanoma and T cell lymphoma, whereas transgenic overexpression enhances rejection of metastasis in mice [11–15]. Recently, a fundamental role of NKp46 in the recognition and subsequent killing of ecto-calreticulin positive ER-stressed cells such as senescent or Zika virus (ZIKV)-infected cells, B16 melanoma, and Ras-driven lung carcinoma cells was detected [16]. Mechanistically, NKp46 recognition of the P domain of ecto-CRT triggers NK cell signaling and enhances tumor-infiltrating NK cell degranulation and cytokine secretion, but the upstream regulators of *Ncr1* remain undefined [16].

NK cells are potent producers of pro-inflammatory cytokines and chemokines that enhance the recruitment and maturation of dendritic cells (DCs) [1, 17, 18]. In parallel, NK cells acquire their functionality through priming by accessory cells, such as DCs, macrophages, monocytes, or neutrophils, which stimulate the maturation and effector activity of NK cells [2, 19–22]. The *trans*-presentation of interleukin-15 (IL-15), critical for the survival and proliferation of NK cells, by the IL-15 receptor α (IL-15Rα) is important as IL-15 enhances NK cell cytotoxicity. Therefore, it is used already in clinical trials, either alone or in combination with other immunotherapies in human cancer patients (https://clinicaltrials.gov/search?cond=Cancer&intr=IL-15) [2, 19–23].

The orphan nuclear receptor subfamily 2, group F, member 6 (NR2F6, EAR2, COUP-TF III) belongs to the nuclear receptor (NR) family, which governs both pro-and anti-inflammatory processes [24, 25]. Network analysis representing TF downstream targets in human NK cells depicts NR2F6 at the core of the BCL11B and RUNX2 gene network [26]. We have been the first to establish an essential and non-redundant functional role of NR2F6 in T lymphocytes as an intracellular immune checkpoint during experimental autoimmunity, bacterial infection, and cancer immune surveillance and metastasis [27–33]. However, a functional investigation of NR2F6 in NK cells has not yet been performed.

## Results

### NKp46 expression within peripheral *Nr2f6*-deficient NK cells is highly enhanced

**Fig. 1:**
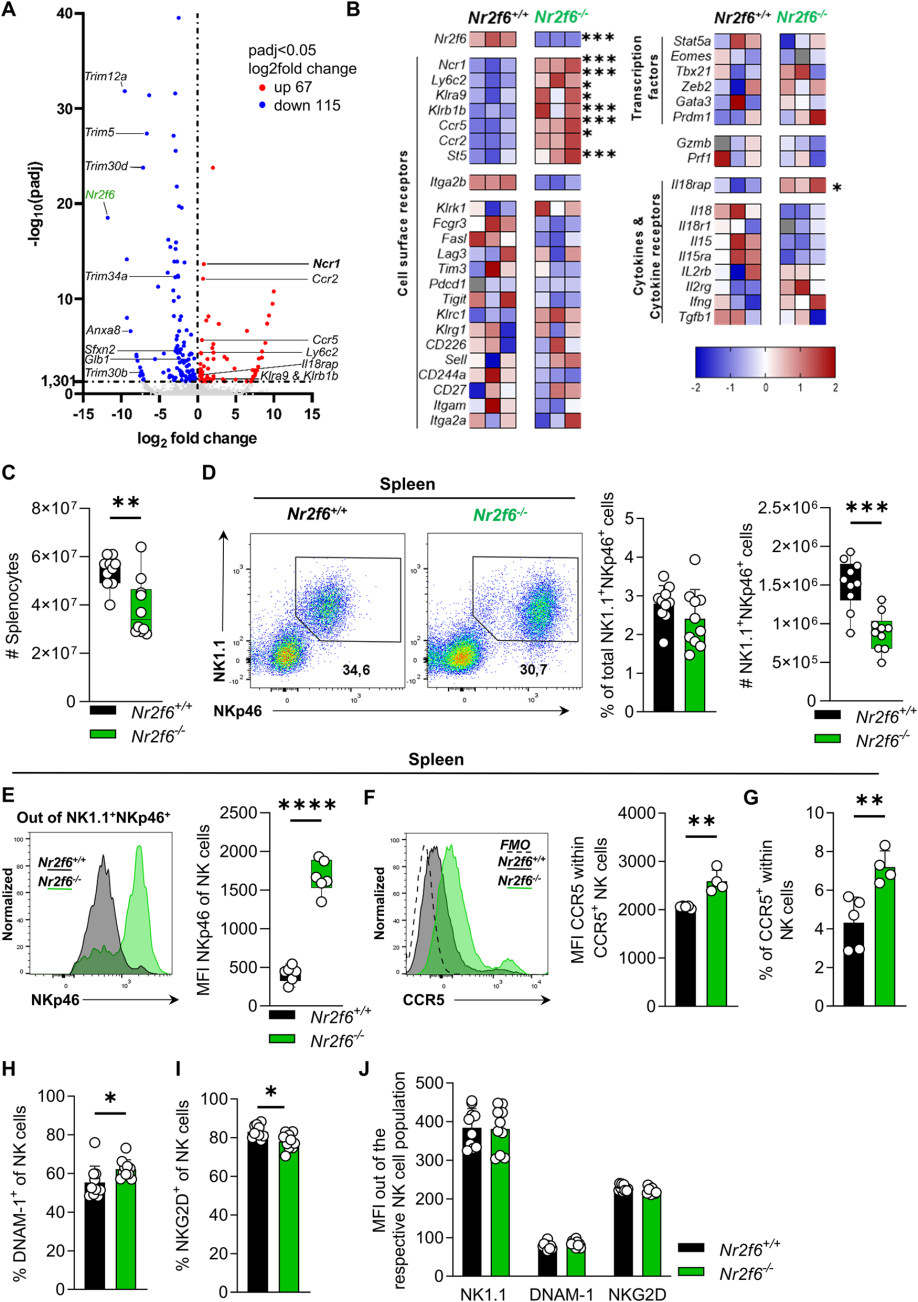
Characterization of *Nr2f6*-deficient NK cells via RNA-Seq analysis. **A** Volcano plot of differentially expressed genes (DEGs) between wild-type (*Nr2f6*^*+/+*^) or *Nr2f6*-deficient (*Nr2f6* ^*−/−*^) splenic NK cells. Genes were considered DEG if the adjusted *p* value (padj) after DESeq2 normalization was <0.05. Downregulated genes are depicted in blue, and upregulated genes in red, NK cell-relevant genes are labeled (*n* = 3 per genotype). **B** Heatmap of the selected cluster of NK cell-defining genes in mice, as defined by the group of Vivier [34] in wild-type (*Nr2f6*^*+/+*^) or *Nr2f6*-deficient (*Nr2f6*^*−/−*^) splenic NK cells. All genes were z-score normalized, and DEGs were defined by DESeq2 (adjusted *p* value (padj) < 0.05) (*n* = 3 per genotype). **C** Quantification of total splenocytes in wild-type (*Nr2f6*^*+/+*^) or *Nr2f6*-deficient (*Nr2f6*^*−/−*^) mice. **D** Representative dot plots and quantification of splenic NK cell frequencies and total cell numbers (CD3^-^CD19^-^NK1.1^+^NKp46^+^) from wild-type (*Nr2f6*^*+/+*^) or *Nr2f6*-deficient (*Nr2f6*^*−/−*^) mice. **E** Representative histogram of NKp46 expression and quantification of the MFI of NKp46 in wild-type (*Nr2f6*^*+/+*^) or *Nr2f6*-deficient (*Nr2f6*^*−/−*^) splenic NK cells (CD3^-^CD19^-^NK1.1^+^NKp46^+^). **F** Representative histogram of CCR5 expression and quantification of the MFI of CCR5 in wild-type (*Nr2f6*^*+/+*^) or *Nr2f6*-deficient (*Nr2f6*^*−/−*^) splenic NK cells (CD3^-^CD19^-^NK1.1^+^NKp46^+^). **G** Quantification of the frequencies of CCR5^+^ splenic NK cells (CD3^-^CD19^-^NK1.1^+^NKp46^+^) in wild-type (*Nr2f6*^+/+^) or *Nr2f6*-deficient (*Nr2f6*^*−/−*^) mice. **H** Quantification of the frequencies of DNAM-1^+^ splenic NK cells (CD3^-^CD19^-^NK1.1^+^NKp46^+^) in wild-type (*Nr2f6*^+/+^) or *Nr2f6*-deficient (*Nr2f6*^*−/−*^) mice. **I** Quantification of the frequencies of NKG2D^+^ splenic NK cells (CD3^-^CD19^-^NK1.1^+^NKp46^+^) in wild-type (*Nr2f6*^+/+^) or *Nr2f6*-deficient (*Nr2f6*^*−/−*^) mice. **J** Quantification of the MFI of NK1.1, DNAM-1 and NKG2D in wild-type (*Nr2f6*^*+/+*^) or *Nr2f6*-deficient (*Nr2f6*^*−/−*^) splenic NK cells (CD3^-^CD19^-^NK1.1^+^NKp46^+^). **A**, **B** RNA sequencing and all downstream analyses were performed on splenic NK cells from *n* = 3 per genotype. **C**, **D**, **H**–**J** Representative data is shown as pooled experiments of at least three independent experiments *n* = 10. **E** Representative data is shown as one of three independent experiments with *n* = 5 per genotype and experiment. **F** Representative data is shown as one of three independent experiments with *n* = 5 wild-type (*Nr2f6*^+/+^) and *n* = 4 *Nr2f6*-deficient (*Nr2f6*^*−/−*^) mice. Each dot represents the data of an individual mouse. Results are shown as mean ± SD. The normality of data was evaluated by the Shapiro–Wilk test. An asterisk indicates statistically significant differences between genotypes calculated using Student’s *t*-test. A *p* value < 0.05 was considered statistically significant. *p < 0.05; **p < 0.01; ***p < 0.001 ****p < 0.0001.

To explore the NR2F6-regulated transcriptional landscape in NK cells under steady-state conditions, we sorted splenic CD3^-^NK1.1^+^NKp46^+^CD49b^+^ NK cells from healthy wild-type and germline *Nr2f6*-deficient mice and performed RNA-Seq (Fig. 1A, S1A-C). Notably, the expression of several genes within the cluster of splenic NK cell-defining genes [34] in mice displayed enhanced expression in *Nr2f6*-deficient NK cells. This cluster included the activating receptor *Ncr1* (NKp46); the IL-18 coreceptor (*Il18rap*); the cell membrane proteins, lymphocyte antigen 6 family memberC2 (*Ly6c2*) and the killer cell lectin-like receptor subfamily A, member 9 (*Klra9*); as well as the inhibitory receptor, killer cell lectin-like receptor subfamily B, member 1B (*Klrb1b*) (Fig. 1A, B). Additionally, the expression of two chemokine receptor genes, C-C motif chemokine receptor (*Ccr*) 2 and *Ccr5*, were significantly enhanced in *Nr2f6*-deficient NK cells, along with DENN domain containing 2B (St5) (Fig. 1A, B).

Simultaneously, we investigated downregulated genes. In parallel to *Nr2f6*, the expression of various family members of the tripartite motif-containing Trim family (*Trim 12a, Trim5, Trim 30* *d*&*b*, and *Trim34a*), as well as Annexin A8 (*Anxa8*); Sideroflexin 2 (*Sfxn2*) and galactosidase, beta 1 (*Glb1*) was reduced (Fig. 1A, B). Gene Set Enrichment Analysis (GSEA) and Kyoto Encyclopedia of Genes and Genomes (KEGG) pathway analysis were conducted, yet no upregulated gene sets were identified.

We investigated the protein levels of RNA-Seq candidates, including NKp46 and CCR5, along with other essential NK cell markers such as NK1.1, NKG2D, or DNAM-1 (CD226) via flow cytometry (Fig. S1D). Of note, splenic cell numbers of *Nr2f6*-deficient mice were generally decreased therefore, a substantial reduction in total cell numbers of NK1.1^+^NKp46^+^, NKG2D^+^, and DNAM-1^+^ NK cells was observed in *Nr2f6*-deficient mice (Fig. 1C, D, S1E, F). Consistent with our RNA-Seq dataset, the expression levels of NKp46 (determined as mean fluorescent intensity (MFI)) and CCR5 were enhanced, as were the frequencies of CCR5 expressing splenic *Nr2f6*-deficient NK cells when compared to wild-type controls (Fig. 1E–G).

The frequencies of DNAM-1 expressing splenic *Nr2f6*-deficient NK cells were slightly enhanced, whereas NKG2D frequencies were marginally lower, but the MFI of NK1.1, DNAM-1, and NKG2D were not altered when compared to wild-type controls (Fig. 1H–J).

**Fig. 2:**
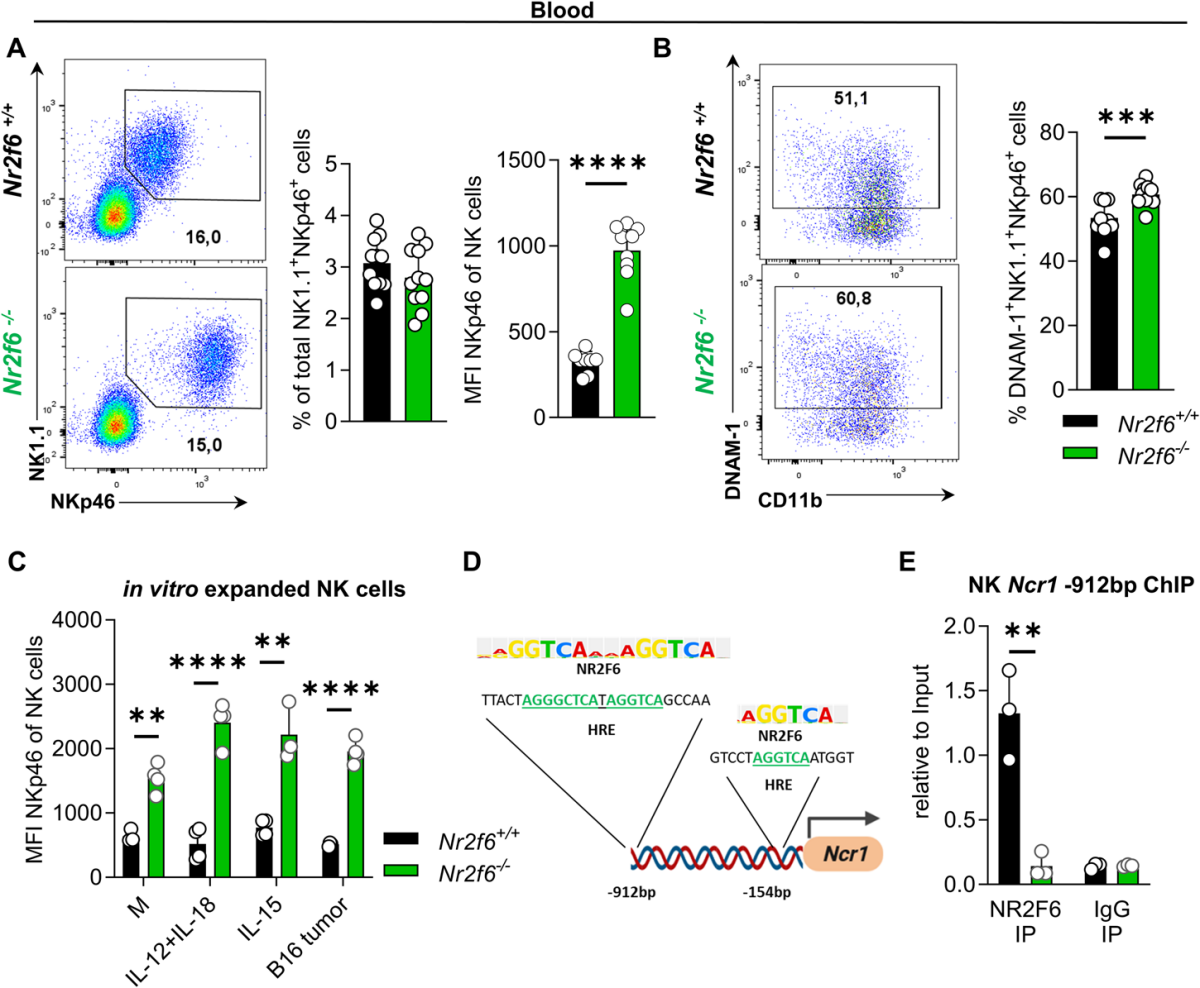
NKp46 expression within *Nr2f6*-deficient NK cells in the blood is highly enhanced. **A** Representative dot plots, quantification of total cell numbers and NKp46 expression (MFI) of blood-derived NK cells (CD3^-^CD19^-^NK1.1^+^NKp46^+^) from wild-type (*Nr2f6*^+/+^) or *Nr2f6*-deficient (*Nr2f6*^*−/−*^) mice. **B** Representative dot plots and quantification of percent of DNAM-1^+^ blood NK cells (CD3^-^CD19^-^NK1.1^+^NKp46^+^) from wild-type (*Nr2f6*^+/+^) or *Nr2f6*-deficient (*Nr2f6*^*−/−*^) mice. **C** NKp46 expression (MFI) of isolated splenic NK cells from wild-type (*Nr2f6*^+/+^) or *Nr2f6*-deficient (*Nr2f6*^*−/−*^) mice. NK cells were cultured in vitro for 7 days in the presence of IL-15 and subsequently left unstimulated (M) or stimulated for 5 hours with IL-12 + IL-18, IL-15, or co-cultured with B16-F10 tumor cells. **D** Prediction of putative NR2F6 (NR2F COUP-TF) binding sites of the mouse *Ncr1* promoter based on position weight matrix from the TRANSFAC database [35]. **E** NR2F6 binding to the *Ncr1* promoter at -912bp was investigated by ChIP. *Nr2f6*^*+/+*^ or *Nr2f6*^*−/−*^ sorted splenic NK cells were used with anti-NR2F6 or IgG2b control precipitation, *Ncr1* promoter was quantified by qPCR, data are presented as relative to input. **A**, **B** Representative data is shown as pooled experiments of at least three independent experiments *n* = 11. **C** One of two independent experiments is shown with *n* = 4 per genotype and experiment. **E** Representative data is shown as pooled experiments of three independent experiments with *n* = 1 per genotype and experiment. Each dot represents the data of an individual mouse. Results are shown as mean ± SD. The normality of data was evaluated by the Shapiro-Wilk test. An asterisk indicates statistically significant differences between genotypes calculated using Student’s *t*-test. A *p* value < 0.05 was considered statistically significant. **p < 0.01***p < 0.001; ****p < 0.0001.

Along this line, in the blood, the expression of NKp46 but also the frequencies of DNAM-1^+^ *Nr2f6*-deficient NK cells were significantly elevated compared to wild-type controls (Fig. 2A, B). To investigate if NKp46 expression is cell-autonomously regulated by NR2F6, wild-type or *Nr2f6*-deficient splenic NK cells were isolated and cultured in vitro for 7 days in the presence of IL-15. Subsequently, the NK cells were stimulated for 5 hours under different conditions (resting, IL-12 + IL-18, IL-15, B16-melanoma cells). Regardless of the stimuli, *Nr2f6*-deficient NK cells consistently exhibited elevated NKp46 levels compared to wild-type controls (Fig. 2C).

To decipher potential NR2F (COUP) DNA binding sites (TGACCT) within the murine *Ncr1* promoter, we employed the TRANSFAC database, which identified two putative binding sites at 912 and 154 base pairs upstream of the *Ncr1* transcription start site (Fig. 2D) [35]. We performed chromatin immunoprecipitation (ChIP) assays in isolated splenic NK cells, utilizing either anti-NR2F6 or IgG-control antibodies and confirmed functional relevance at the −912 site within the promoter locus (Fig. 2E).

In summary, loss of NR2F6 significantly enhances NKp46 expression at the transcriptional and surface receptor levels in peripheral splenic and blood-derived NK cells. ChIP results provide evidence supporting the regulatory function of NR2F6 in suppressing murine *Ncr1* gene expression.

### NKp46 expression is enhanced in bone marrow-derived *Nr2f6*-deficient NK cells

**Fig. 3:**
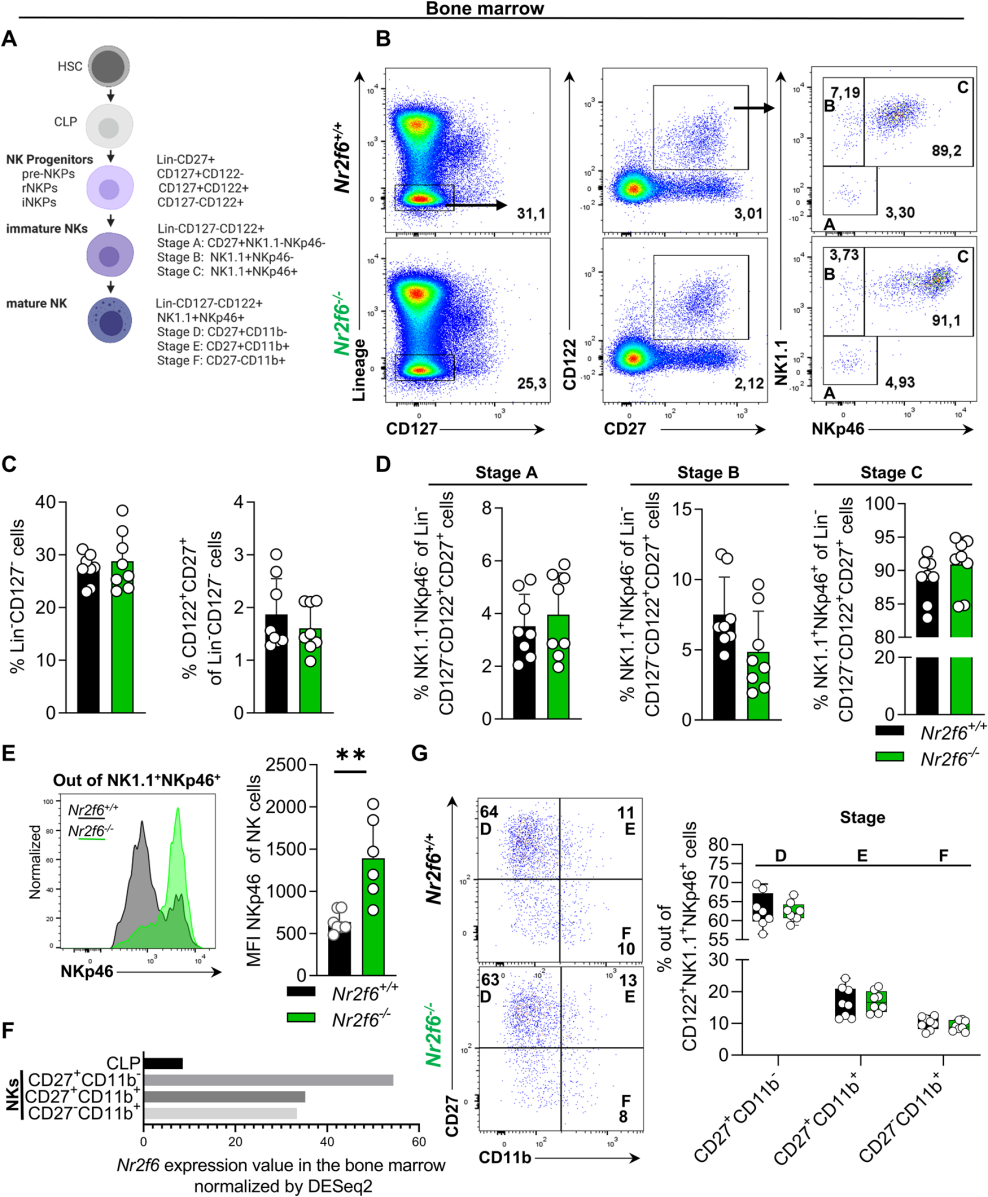
*Nr2f6*-deficient mice have normal NK cell development in the bone marrow, NKp46 expression is increased. **A** Schematic representation of murine NK cell development in the bone marrow arising from hematopoietic stem cells (HSCs) via common lymphoid progenitors (CLPs). **B** Representative dot plots of bone marrow derived Lin^-^CD127^-^ and immature (Lin^-^CD127^-^CD122^+^CD27^+^, NK1.1^-^NKp46^-^, NK1.1^+^NKp46^-^, NK1.1^-^NKp46^-^) NK cell populations from wild-type (*Nr2f6*^+/+^) or *Nr2f6*-deficient (*Nr2f6*^*−/−*^) mice. **C** Quantification of the frequency of parent in bone marrow-derived Lin^-^CD127^-^ cells and immature NK cells (Lin^-^CD127^-^CD122^+^CD27^+^) in wild-type (*Nr2f6*^+/+^) or *Nr2f6*-deficient (*Nr2f6*^*−/−*^) mice. **D** Quantification of the frequency in bone marrow-derived Lin^-^CD127^-^CD122^+^CD27^+^ stage A (NK1.1^-^NKp46^-^) stage B (NK1.1^+^NKp46^-^) and stage C (NK1.1^+^NKp46^+^) NK cells in wild-type (*Nr2f6*^+/+^) or *Nr2f6*-deficient (*Nr2f6* ^*−/−*^) mice. **E** Representative histogram of NKp46 expression and quantification of the MFI of NKp46 in Lin^-^CD127^-^CD122^+^CD27^+^NK1.1^+^NKp46^+^ wild-type (*Nr2f6*^+/+^) or *Nr2f6*-deficient (*Nr2f6*^*−/−*^) NK cells. **F** *Nr2f6* expression from common lymphoid progenitors (CLPs) (CD93^+^CD117^+^IL7Ra^+^CD45R^-^) and maturing NK cell populations (CD27^+^CD11b^-^, CD27^+^CD11b^+^, CD27^-^CD11b^+^ out of CD3^-^CD19^-^NK1.1^+^CD127^-^CD51^-^CD49a^-^DX5^+^) in the bone marrow, normalized by DESeq2 based on the Immgen consortium database [37]. **G** Representative dot plot and quantification of bone marrow-derived maturing NK cell populations stage D (CD27^+^CD11b^-^), stage E (CD27^+^CD11b^+^) and stage F (CD27^-^CD11b^+^) out of Lin^-^CD127^-^CD122^+^NK1.1^+^NKp46^+^ NK cells in wild-type (*Nr2f6*^+/+^) or *Nr2f6*-deficient (*Nr2f6*^*−/−*^) mice. **C**, **D**, **F**, **G** Representative data is shown as pooled experiments of at least three independent experiments *n* = 8. (**E** Representative data is shown as pooled experiments of two independent experiments *n* = 7 wild-type (*Nr2f6*^+/+^) and *n* = 6 *Nr2f6*-deficient (*Nr2f6*^*−/−*^) mice. Each dot represents the data of an individual mouse. Results are shown as mean ± SD. The normality of data was evaluated by the Shapiro–Wilk test. An asterisk indicates statistically significant differences between genotypes calculated using Student’s *t*-test. A *p* value < 0.05 was considered statistically significant.**p < 0.01.

To investigate the onset of NKp46 expression during NK cell development in the bone marrow, together with NK cell precursors, we analyzed progenitor populations in healthy wild-type and *Nr2f6*-deficient mice. The frequencies and total cell numbers of common lymphoid progenitors (CLP), pre-NK precursor (NKPs), and refined NK precursor (rNKPs) were similar between genotypes (Fig. 3A-C; S2A-C). During NK cell development, immature NK (iNK) cells pass through three developmental stages [36]. Percentages and cell numbers of stages A, B, and C were similar between the genotypes, however, upon reaching stage C, characterized by the upregulation of NKp46, *Nr2f6*-deficient CD3^-^NK1.1^+^ cells exhibited significantly elevated levels of NKp46 expression (Fig. 3B, D, E, S2D). Along this line, it is worth noting that *Nr2f6* expression is barely detectable in CLP; but gets upregulated during NK cell development (Fig. 3F) [37]. Further characterization of the maturation of NK (mNK) cells in the BM from immature CD27^+^CD11b^-^, CD27^+^CD11b^+^, to mature CD27^-^CD11b^+^ did not reveal differences between genotypes (Fig. 3G). Taken together, in the BM, loss of NR2F6 does not alter NK cell progenitor populations or maturation but enhances NKp46 expression upon induction.

### The loss of NR2F6 blocks peripheral NK cell maturation and short-term effector responses

**Fig. 4:**
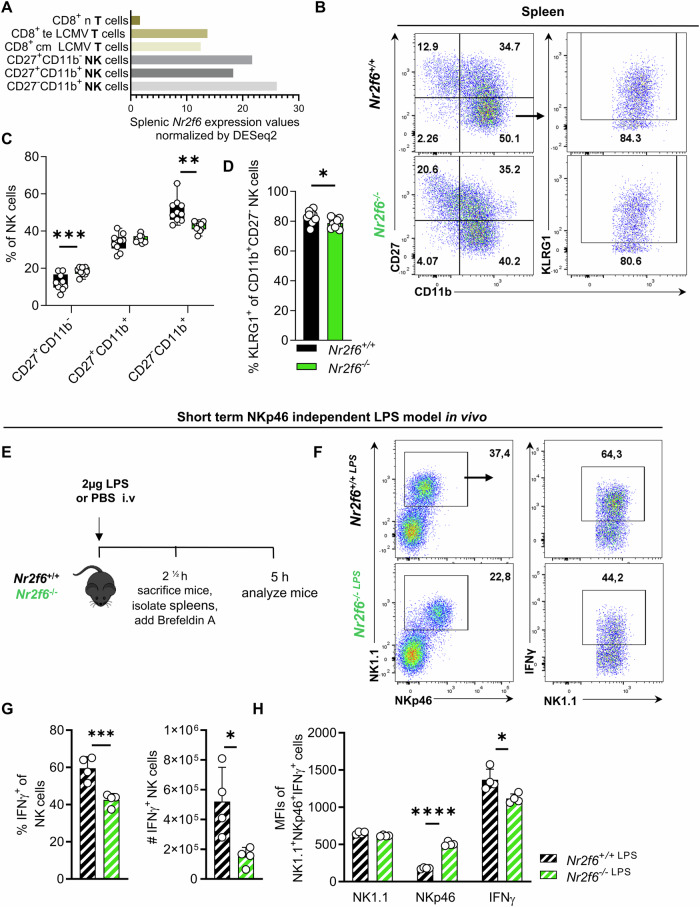
Terminal NK cell maturation block in *Nr2f6*-deficient mice and reduced NKp46 independent short-term effector responses. **A** *Nr2f6* expression values in splenic CD8 T cell subsets, naïve (n), effector (te), and central-memory (cm) on day 7 post lymphocytic choriomeningitis virus (LCMV) infection, and the different splenic natural killer (NK) cell subsets CD27^+^CD11b^-^, CD27^+^CD11b^+^, and CD27^-^CD11b^+^. Expression values are normalized by DESeq2 based on the Immgen consortium database [37]. **B** Representative dot-plots of CD27^-^CD11b^-^, CD27^+^CD11b^-^, CD27^+^CD11b^+^ mature CD27^-^CD11b^+^, and terminally matured (CD27^-^CD11b^+^KLRG1^+^) splenic NK cell populations (out of CD45^+^CD3^-^NK1.1^+^NKp46^+^) in wild-type (*Nr2f6*^+/+^) or *Nr2f6*-deficient (*Nr2f6*^*−/−*^) mice. **C** Quantification of frequency CD27^+^CD11b^-^, CD27^+^CD11b^+^ and mature CD27^-^CD11b^+^ splenic NK cells (CD45^+^CD3^-^NK1.1^+^NKp46^+^) in wild-type (*Nr2f6*^+/+^) or *Nr2f6*-deficient (*Nr2f6*^*−/−*^) mice. **D** Quantification of terminally matured KLRG1^+^ NK cells (CD45^+^CD3^-^NK1.1^+^NKp46^+^ CD27^-^CD11b^+^) in the spleen of wild-type (*Nr2f6*^+/+^) or *Nr2f6*-deficient (*Nr2f6*^*−/−*^) mice. **E** Schematic overview of the experimental setup of LPS injection. Wild-type (*Nr2f6*^+/+^) or *Nr2f6* deficient (*Nr2f6* ^*−/−*^) mice were i.v. injected with 2 µg LPS or PBS as control and the animals were sacrificed 2 ½ hrs later. Splenocytes were isolated and incubated for another 2 ½ hrs in the presence of Brefeldin A. **F** Representative dot-plots of splenic NK cells (CD45^+^CD3^-^NK1.1^+^NKp46^+^) IFNγ-producing NK cells in wild-type (*Nr2f6*^+/+^) or *Nr2f6*-deficient (*Nr2f6*^*−/−*^) mice after LPS injection. **G** Quantification of frequencies and total cell counts of IFNγ^+^ NK cells (CD45^+^CD3^-^NK1.1^+^NKp46^+^) in wild-type (*Nr2f6*^+/+^) or *Nr2f6*-deficient (*Nr2f6*^*−/−*^) mice after LPS injection. **H** Quantification of NK1.1, NKp46, and IFNγ expression (MFI) in NK cells (CD45^+^CD3^-^NK1.1^+^NKp46^+^) in wild-type (*Nr2f6*^+/+^) or *Nr2f6*-deficient (*Nr2f6*^*−/−*^) mice after LPS injection. **B**-**D** Representative data is shown as pooled experiments of at least two independent experiments *n* = 8-10. **E**, **F** For LPS injection, the representative data shown are from one independent out of two replicative experiments, with *n* = 4 per group and experiment. Each dot represents the data of an individual mouse. Results are shown as mean ± SD. The normality of data was evaluated by the Shapiro–Wilk test. An asterisk indicates statistically significant differences between genotypes calculated using Student’s *t*-test. A *p* value < 0.05 was considered statistically significant. *p < 0.05; **p < 0.01; ***p < 0.001; ****p < 0.0001.

*Nr2f6* expression is increased in splenic NK cell subsets compared to naïve, effector, and memory CD8 T cell subsets in the spleen, in which the role of *Nr2f6* has already been well characterized (Fig. 4A) [27, 29, 32, 37]. Upon analyzing the maturation status of *Nr2f6*-deficient NK cells in both the spleen and blood, unexpectedly, and in contrast to the bone marrow, we observed an over-representation of immature NK cells, coupled with a significant decrease in the frequencies of mature and terminally matured (CD27^-^CD11b^+^KLRG1^+^) NK cell populations compared to wild-type controls (Figs. 4B–D; S3A-C). Along this line the MFI of CD27 was enhanced, whereas the MFI of CD11b was reduced when compared to wild-type controls (Fig. S3D).

Next, we aimed to assess *Nr2f6*-deficient splenic NK cell effector responses in a model independently of NKp46 activation. We employed the in vivo short-term LPS-driven inflammation model established by the group of Granucci (Fig. 4E) [2]. Following LPS injection, DCs are activated and secrete IL-2, IL-18, and IFN-β, which induces NK cell activation. Although IFNγ production in NK cells was robustly induced in both genotypes, the frequencies, cell numbers, and IFNγ expression (MFI) were significantly reduced in *Nr2f6*-deficient NK1.1^+^NKp46^+^ NK cells when compared to wild-type (Fig. 4E-H; S3E). Notably, the MFI of NKp46 remained elevated in *Nr2f6*-deficient NK cells (Fig. 4F, H).

Taken together, in contrast to the bone marrow, *Nr2f6*-deficient peripheral NK cell maturation in the spleen and the blood and short-term effector response are decreased.

### A comprised myeloid compartment blocks peripheral NK cell maturation in *Nr2f6*-deficient mice

**Fig. 5:**
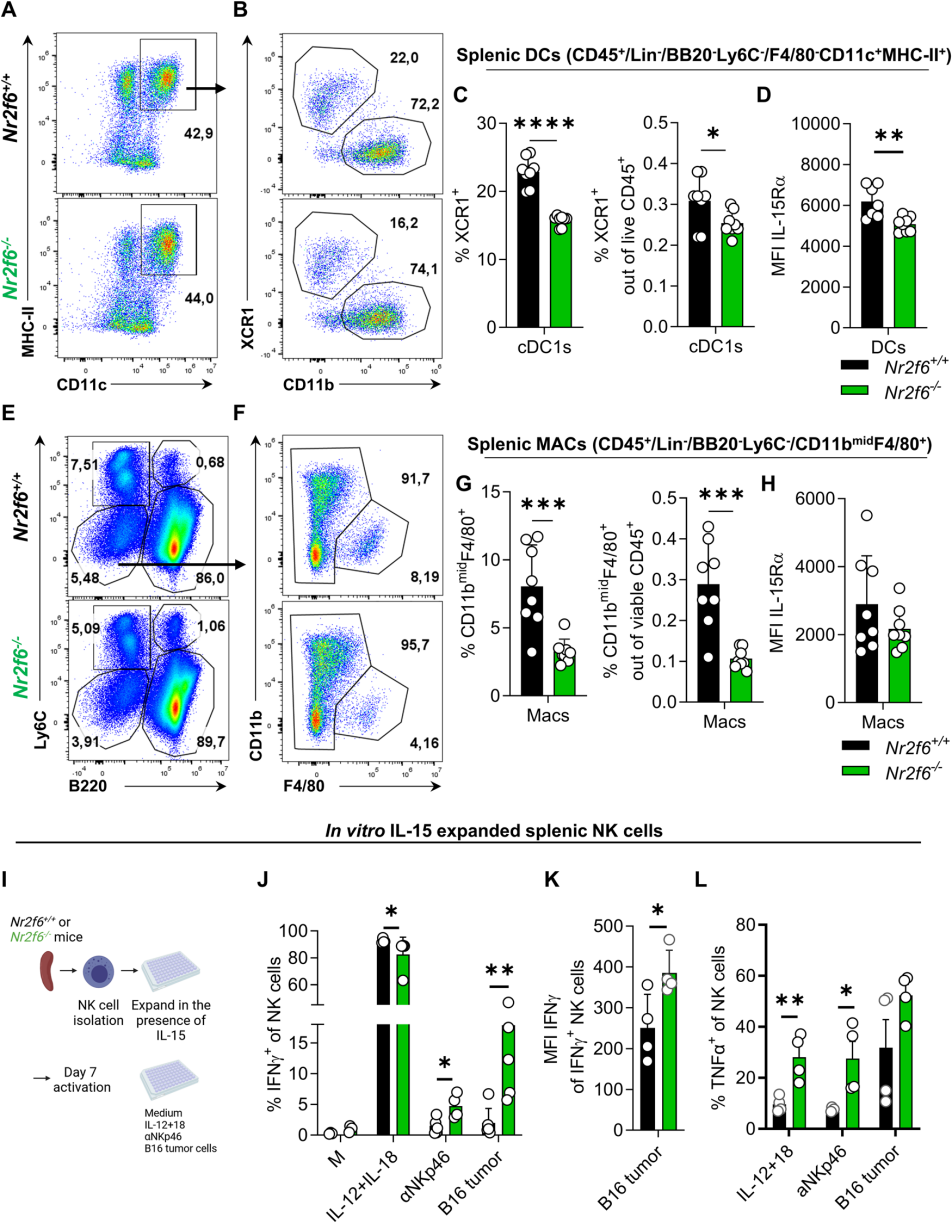
Loss of NR2F6 reduces the splenic cDC1 and macrophage compartment. **A** Representative dot-plots of splenic DC (CD11c^+^MHC-II^+^) populations (out of CD45^+^Lin^-^BB20^-^Ly6C^-^F4/80^-^) in wild-type (*Nr2f6*^+/+^) or *Nr2f6*-deficient (*Nr2f6*^*−/−*^) mice. **B** Representative dot-plots of splenic XCR1^+^cDC1 (XCR1^+^CD11b^-^) populations derived from CD45^+^Lin^-^BB20^-^Ly6C^-^F4/80^-^ CD11c^+^MHC-II^+^ cells in wild-type (*Nr2f6*^+/+^) or *Nr2f6*-deficient (*Nr2f6*^*−/−*^) mice. **C** Quantification of the frequencies of parent and of CD45^+^ splenic XCR1^+^cDC1 cells in wild-type (*Nr2f6*^+/+^) or *Nr2f6*-deficient (*Nr2f6*^*−/−*^) mice. **D** Quantification of IL-15Rα MFI in splenic DC populations in wild-type (*Nr2f6*^+/+^) or *Nr2f6*-deficient (*Nr2f6*^*−/−*^) mice. **E** Representative dot-plots of splenic Ly6C^-^B220^-^, monocyte (Ly6C^+^B220^-^), plasmacytoid DC (Ly6C^+^B220^+^), and B cell (Ly6C^-^B220^+^) populations in wild-type (*Nr2f6*^+/+^) or *Nr2f6*-deficient (*Nr2f6*^*−/−*^) mice. **F** Representative dot-plots of splenic macrophage (CD11b^mid^F4/80^+^) populations derived from Ly6C^-^B220^-^ in wild-type (*Nr2f6*^+/+^) or *Nr2f6*-deficient (*Nr2f6*^*−/−*^) mice. **G** Quantification of frequency of parent and of CD45^+^ total splenic macrophages (CD45^+^Lin^-^B220^-^Ly6C^-^CD11b^mid^F4/80^+^) in wild-type (*Nr2f6*^+/+^) or *Nr2f6*-deficient (*Nr2f6*^*−/−*^) mice. **H** Quantification of IL-15Rα expression (MFI) in splenic macrophage populations of wild-type (*Nr2f6*^+/+^) or *Nr2f6*-deficient (*Nr2f6*^*−/−*^) mice. **I** Schematic overview of the experimental setup, splenic NK cells of wild-type (*Nr2f6*^+/+^) or *Nr2f6*-deficient (*Nr2f6*^*−/−*^) mice were isolated and expanded in vitro for 7 days with 50 ng/ml IL-15. NK cells were left unstimulated (**M**) or were stimulated for 5 hours with IL-12 + IL-18, α-NKp46, or co-cultured with B16-F10 tumor cells, IFNγ and TNFα cytokine levels were measured. **J** Frequency of IFNγ producing NK cells, **K** MFI of IFNγ expressing NK cells and **L** frequency of TNFα producing NK cells were quantified for wild-type (*Nr2f6*^+/+^) or *Nr2f6*-deficient (*Nr2f6*^*−/−*^) NK cells. **A**–**H** Representative data are shown as pooled experiments of two independent experiments *n* = 8. **J**–**L** The representative data shown are from one independent experiment out of two replicative experiments, with *n* = 4 per group and experiment. Each dot represents the data of an individual mouse. Results are shown as mean ± SD. The normality of data was evaluated by the Shapiro–Wilk test. An asterisk indicates statistically significant differences between genotypes calculated using Student’s *t*-test, or Mann–Whitney *U* test. A *p* value < 0.05 was considered statistically significant. *p < 0.05; **p < 0.01; ***p < 0.001; ****p < 0.0001.

NK cells acquire full maturation potential and functionality through priming by accessory myeloid cells such as DCs, macrophages, or monocytes [19–22]. We characterized the splenic myeloid compartment to pinpoint the eventual NK cell-extrinsic factor missing for priming *Nr2f6*-deficient NK cells in vivo (Fig. S4A). Our analysis revealed significantly reduced frequencies of the cross-presenting XCR1^+^cDC1s both on a percentage of parent and within living CD45^+^ cells compared to wild-type controls (Fig. 5A–C). Given that NK cell maturation relies on IL-15 cross-presentation by accessory myeloid cells, we further explored the expression (MFI) of IL-15Rα [21, 22]. Our analysis revealed lower IL-15Rα on *Nr2f6*-deficient DCs than wild-type controls (Fig. 5D).

The *Nr2f6*-deficient CD11b^lo-mid^F4/80^+^ macrophage compartment was also strongly reduced both in terms of frequencies of the parent population and when calculated per live CD45^+^ cells, but the expression of IL-15Rα on *Nr2f6*-deficient macrophages was similar to wild-type (Fig. 5E–H).

To investigate the role of IL-15 during NK cell priming in vitro, we cultured isolated splenic NK cells with IL-15 for one week (Fig. 5I). In vitro expanded *Nr2f6*-deficient NK cells decreased IFNγ and TNFα responses upon stimulation with IL12 + IL-18, but increased following NKp46 activation compared to wild-type controls. Furthermore, when co-cultured with B16 tumor cells, *Nr2f6*-deficient NK cells exhibited significantly enhanced IFNγ production in percentages and MFI (Fig. 5J–L). Therefore, a comprised splenic myeloid compartment reduces IL-15 priming of NK cells and, subsequently, maturation and effector responses in *Nr2f6*-deficient mice.

### Overcoming IL-15 limitation in vivo enables *Nr2f6*-deficient NK cells to develop their full maturation potential

**Fig. 6:**
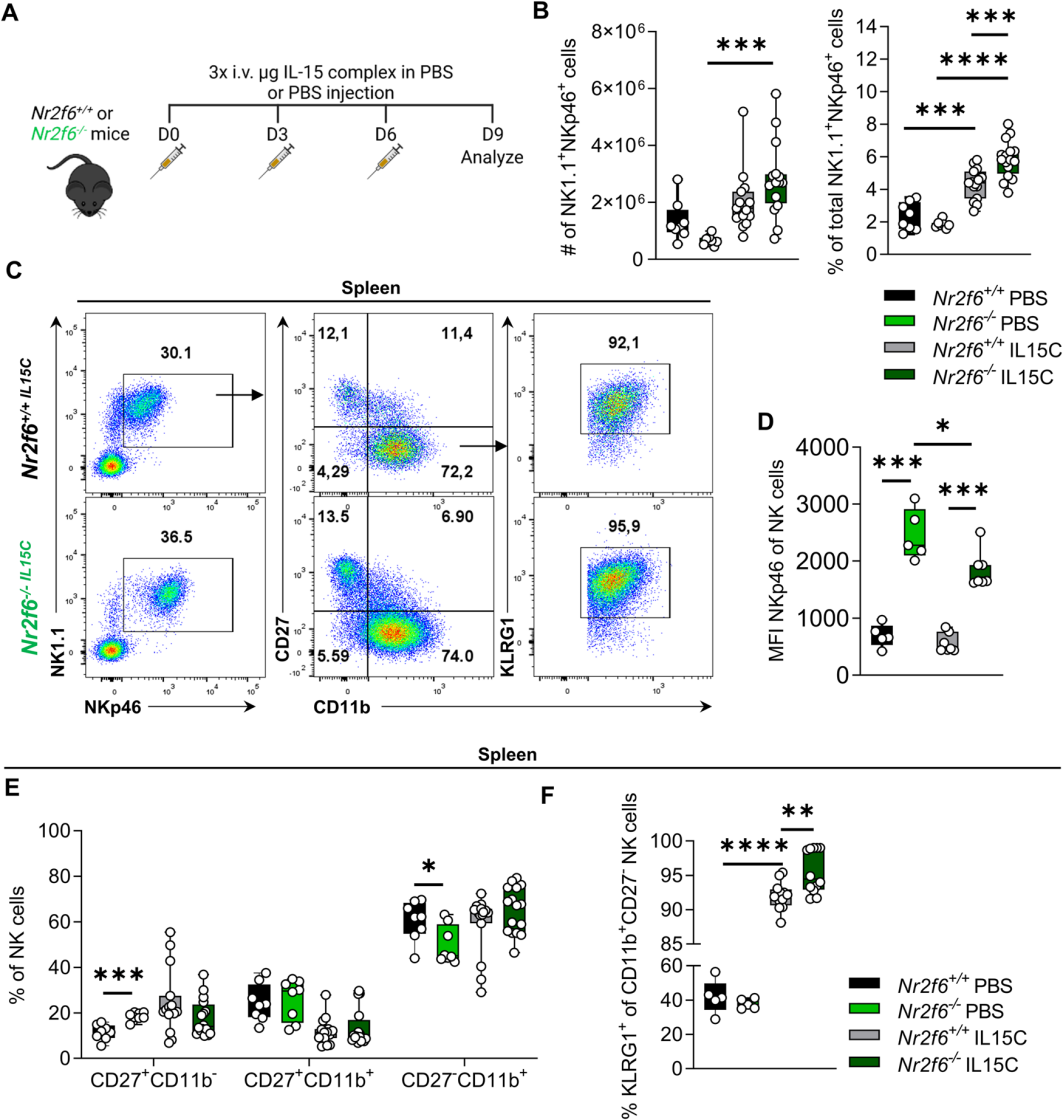
IL-15 complex treatment restores NK maturation. **A** Schematic overview of the experimental setup of IL-15/IL-15Rα-Fc treatment. Wild-type (*Nr2f6*^+/+^) or *Nr2f6*-deficient (*Nr2f6*^*−/−*^) mice were injected 3 x with IL-15/IL-15Rα-complex or PBS i.v. Mice were analyzed nine days after the first injection. **B** Quantification of total cell counts and frequencies of splenic NK cells (CD45^+^CD3^-^CD19^-^NK1.1^+^NKp46^+^) in wild-type (*Nr2f6*^+/+^) or *Nr2f6*-deficient (*Nr2f6*^*−/−*^) mice after IL-15/IL-15Rα-Fc or PBS injection. **C** Representative dot-plots of total splenic NK cells (CD45^+^CD3^-^CD19^-^NK1.1^+^NKp46^+^), maturation (CD27^-^CD11b^-^, CD27^+^CD11b^-^, CD27^+^CD11b^+^, CD27^-^CD11b^+^), and terminally matured (CD27^-^CD11b^+^ KLRG1^+^) NK cell populations in wild-type (*Nr2f6*^+/+^) or *Nr2f6*-deficient (*Nr2f6*^*−/−*^) mice after IL-15/IL-15Rα-Fc injection. **D** Quantification of NKp46 expression (MFI) in splenic NK cells (CD45^+^CD3^-^CD19^-^NK1.1^+^NKp46^+^) of wild-type (*Nr2f6*^+/+^) or *Nr2f6*-deficient (*Nr2f6*^*−/−*^) mice after IL-15/IL-15Rα-Fc or PBS injection. **E** Quantification of frequencies of CD27^+^CD11b^-^, CD27^+^CD11b^+^ and mature CD27^-^CD11b^+^ splenic NK cell (CD45^+^CD3^-^CD19^-^NK1.1^+^NKp46^+^) in wild-type (*Nr2f6*^+/+^) or *Nr2f6*-deficient (*Nr2f6*^*−/−*^) mice after IL-15/IL-15Rα-Fc or PBS injection. **F** Quantification of the frequency of terminally matured (CD27^-^CD11b^+^KLRG1^+^) NK cells (CD45^+^CD3^-^CD19^-^NK1.1^+^NKp46^+^) in wild-type (*Nr2f6*^+/+^) or *Nr2f6*-deficient (*Nr2f6*^*−/−*^) mice after IL-15/IL-15Rα-Fc or PBS injection. **B**, **E**–**F** Representative data are shown as pooled experiments of five independent experiments *n* = 8 (PBS treated wild-type (*Nr2f6*^+/+^) or *Nr2f6*-deficient (*Nr2f6*^*−/−*^) mice) and *n* = 15 (IL15C treated wild-type (*Nr2f6*^+/+^) mice) or *n* = 16 (IL-15C treated *Nr2f6*-deficient (*Nr2f6*^*−/−*^) mice). **D** The representative data shown are from one independent experiment out of five replicative experiments, with *n* = 2-4 per group and experiment. Each dot represents the data of an individual mouse. Results are shown as mean ± SD. The normality of data was evaluated by the Shapiro–Wilk test. An asterisk indicates statistically significant differences between genotypes calculated using Student’s *t*-test or Mann-Whitney *U* test. A *p* value < 0.05 was considered statistically significant. *p < 0.05; **p < 0.01; ***p < 0.001; ****p < 0.0001;.

We next assess the potential of IL-15-complex (IL-15C) treatment, which is more potent than IL-15 alone, in rescuing *Nr2f6*-deficient NK cell maturation and effector functions in vivo (Fig. 6A; S5A) [38–40]. Following three times IL-15C injections, total splenic and NK cell numbers increased in *Nr2f6*-deficient mice and were similar to wild-type (Fig. S5B). The frequencies of IL-15C-treated NK cells were especially high in the spleen and blood of *Nr2f6*-deficient mice and strongly enhanced when compared to IL-15C-treated wild-type controls (Figs. 6B; S5C-D). Of importance, the MFI of NKp46 remained significantly elevated in splenic and blood-derived *Nr2f6*-deficient NK cells when compared to wild-type (Figs. 6C, D; S5C, E). IL-15C treatment normalized the maturation of *Nr2f6*-deficient CD11b^+^NK cells in the spleen and blood and significantly increased frequencies of terminally mature CD11b^+^KLRG1^+^ NK cells even surpassing wild-type levels (Fig. 6C, E, F; S5C, F, G).

**Fig. 7:**
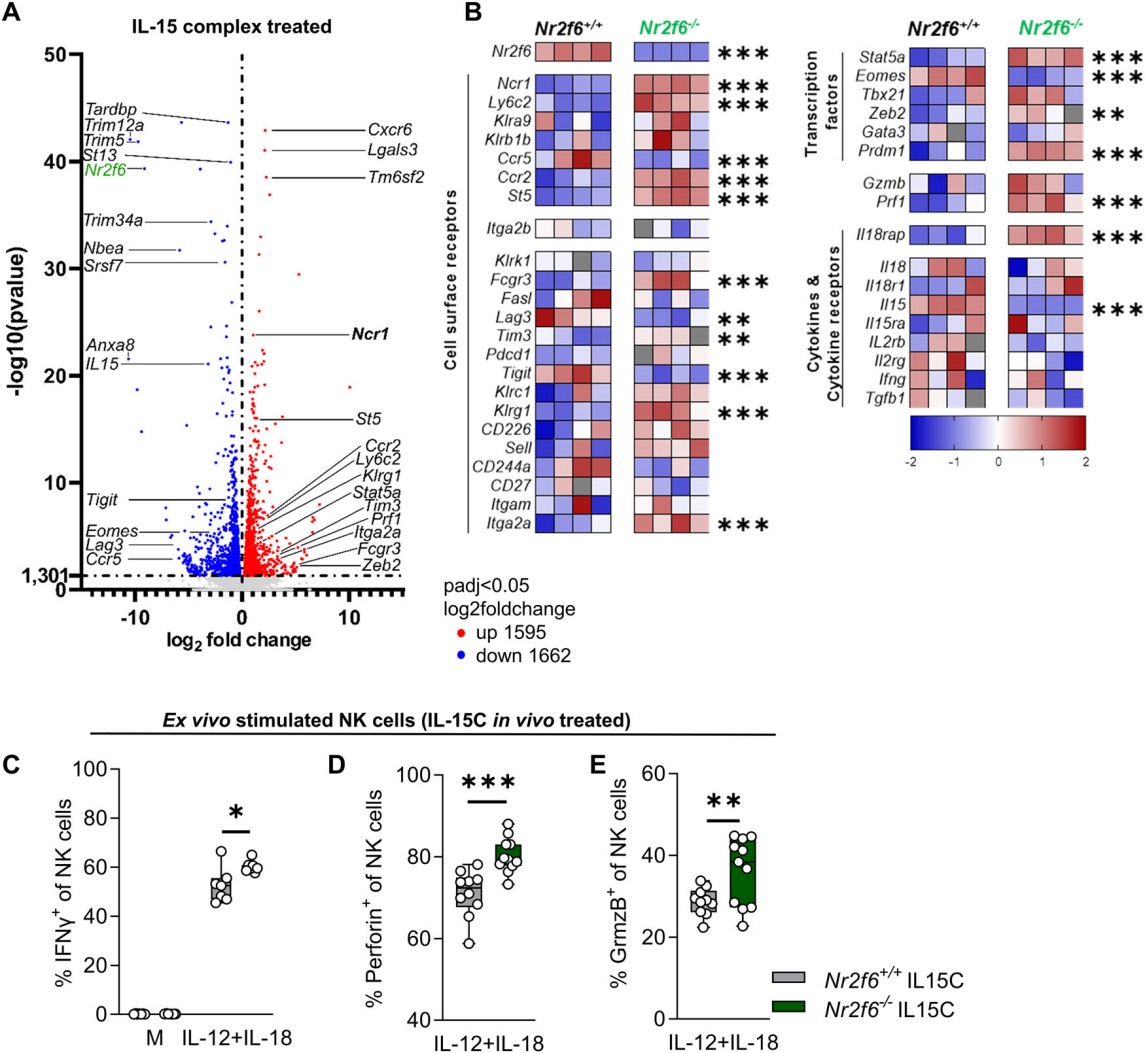
Characterization of IL-15C treated *Nr2f6*-deficient NK cells via RNA-Seq analysis. **A** Volcano plot of differentially expressed genes (DEGs) after IL-15/IL-15Rα-Fc treatment between wild-type (*Nr2f6*^*+/+*^) or *Nr2f6*-deficient (*Nr2f6*^*−/−*^) splenic NK cells. Genes were considered DEG if the adjusted *p* value (padj) after DESeq2 normalization was <0.05. Downregulated genes are depicted in blue, and upregulated genes in red, NK cell-relevant genes are labeled (*n* = 4 per genotype). **B** Heatmap of the selected cluster of NK cell-defining genes in mice, as defined by the group of Vivier [34] in wild-type (*Nr2f6*^*+/+*^) or *Nr2f6*-deficient (*Nr2f6*^*−/−*^) IL-15/IL-15Rα-Fc treated splenic NK cells. All genes were z-score normalized, and DEGs were defined by DESeq2 (adjusted *p* value (padj) < 0.05) (*n* = 4 per genotype). **C** Quantification of frequencies of IFNγ^+^ (**D**), Perforin^+^ (**E**) or Granzyme B^+^ NK cells (CD45^+^CD3^-^CD19^-^NK1.1^+^NKp46^+^). NK cells were isolated from wild-type (*Nr2f6*^+/+^) or *Nr2f6*-deficient (*Nr2f6*^*−/−*^) mice after IL-15/IL-15Rα-Fc injection and ex vivo stimulated for 5 hours with medium alone or medium supplemented with IL-12 and IL-18. **A**, **B** RNA sequencing and all downstream analyses were performed on splenic NK cells from *n* = 4 per genotype. **C** Representative data are shown as pooled experiments of two independent experiments *n* = 7. **D**, **E** Representative data are shown as pooled experiments of three independent *n* = 11. Each dot represents the data of an individual mouse. Results are shown as mean ± SD. The normality of data was evaluated by the Shapiro–Wilk test. An asterisk indicates statistically significant differences between genotypes calculated using Student’s *t*-test. A *p* value < 0.05 was considered statistically significant. *p < 0.05; **p < 0.01; ***p < 0.001.

We sorted NK cells following IL-15C treatment and performed RNA-Seq analysis (Fig. 7A, S1A, B; S6A). In parallel to the steady state conditions, expression of *Ncr1, Ly6c2, Ccr2, St5*, and *Il18ra* were significantly upregulated, whereas *Ccr5* expression was reduced in *Nr2f6*-deficient IL-15C treated NK cells compared to wild-type controls (Fig. 7B). Following IL-15C treatment expression of *Fcgr3, Itga2a*, *Prdm1*, and in line with the flow cytometric data, *Klrg1* mRNA expression was significantly enhanced in *Nr2f6*-deficient IL-15C treated NK cells (Fig. 7B). Furthermore, expression of the TFs *Stat5a*, *Zeb2*, and the effector molecule perforin (*Prf1)* was significantly enhanced. In contrast, the TF *Eomes* and the expression of the inhibitory receptors *Lag3* and *Tigit* were downregulated considerably in *Nr2f6*-deficient NK cells following IL-15C treatment (Fig. 7B).

Furthermore, ex vivo culture of IL-15C treated *Nr2f6*-deficient splenocytes under resting or cytokine stimulatory conditions (IL-12 + IL-18) significantly increased *Nr2f6*-deficient NK cell effector functions measured by the percentage of IFNγ, Perforin, and Granzyme B-producing NK cells, compared to wild-type controls (Fig. 7C–E).

### Loss of NR2F6 protects against B16-F10-*B2m*^*−/−*^ melanoma lung metastasis formation

**Fig. 8:**
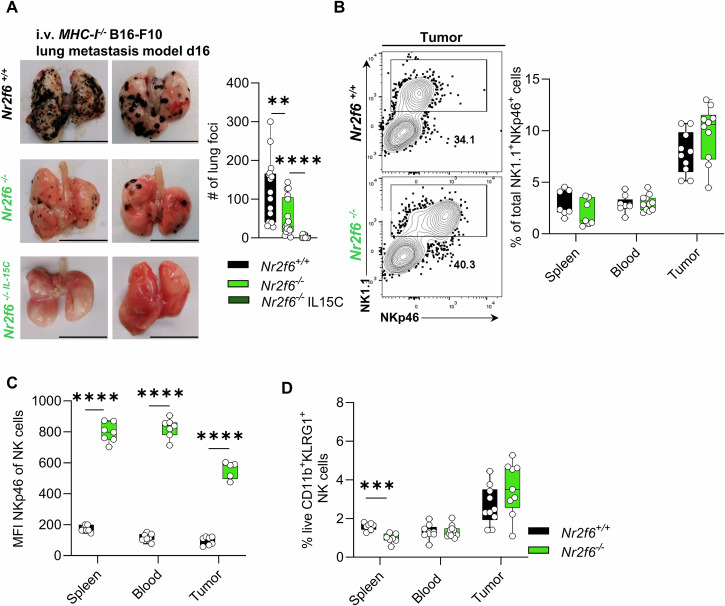
Loss of NR2F6 protects against B16-F10-*B2m*^*−/−*^ lung metastasis formation. **A** Gross examination and quantification of representative metastatic tumor foci in the lung at day 16 after B16-F10-*B2m*^*−/−*^ tumor inoculation of either *Nr2f6*^+/+^ mice or *Nr2f6*^*−/−*^ mice, with or without IL-15C treatment (i.v.-injected). **B** Representative dot-plots (tumor) and quantification of splenic, blood, and lung metastasis-derived NK cell frequencies (CD45^+^CD3^-^CD19^-^NK1.1^+^NKp46^+^) in wild-type (*Nr2f6*^+/+^) or *Nr2f6*-deficient (*Nr2f6*^*−/−*^) mice on day 16 after B16-F10-*B2m*^*−/−*^ tumor injection. **C** Quantification of NKp46 expression (MFI) in splenic, blood, and lung metastasis-derived NK cells (CD45^+^CD3^-^CD19^-^NK1.1^+^NKp46^+^) in wild-type (*Nr2f6*^+/+^) or *Nr2f6*-deficient (*Nr2f6*^*−/−*^) mice on day 16 after B16-F10-*B2m*^*−/−*^ tumor injection. **D** Quantification of the frequency of terminally matured (CD27^-^CD11b^+^KLRG1^+^) NK cells (CD45^+^CD3^-^CD19^-^NK1.1^+^NKp46^+^) in the spleen, the blood and lung metastasis in wild-type (*Nr2f6*^+/+^) or *Nr2f6*-deficient (*Nr2f6*^*−/−*^) mice. **A** Representative data are shown as pooled experiments *n* = 16 (*Nr2f6*^*+/+*^) *n* = 17 (*Nr2f6*^*−/−*^) from five experiments. IL-15C treated *Nr2f6*^*−/−*^ mice are shown as pooled experiments *n* = 10 from two experiments. **B** Representative data are shown as pooled experiments from three independent experiments *n* = 7–11. **D** Representative data are shown as pooled experiments from two independent experiments *n* = 5–7. Each dot represents the data of an individual mouse. Results are shown as mean ± SD. The normality of data was evaluated by the Shapiro–Wilk test. An asterisk indicates statistically significant differences between genotypes calculated using Student’s *t*-test or Mann-Whitney *U* test. A *p* value < 0.05 was considered statistically significant. *p < 0.05; **p < 0.01; ***p < 0.001; ****p < 0.0001.

In healthy mice, only a minor fraction of blood-derived soluble IL-15 is complexed, whereas, in inflammatory settings, most of the circulating IL-15 converts to the soluble complex isoform, thus acting as a rapid alert response [40]. Therefore, we suggested that elevated NKp46 expression could still enhance antitumor NK cell responses in *Nr2f6-*deficient mice during cancer progression. We chose the B16-F10-*B2m*^*−/−*^ (melanoma) tumor metastasis model, which is CD8 T cell-independent, established by the group of Raulet and others [7, 41]. Upon intravenous injection of 2.5×10^5^ B16-F10-*B2m*^*−/−*^ cells, metastasis formation on day 16 was significantly reduced in *Nr2f6*-deficient mice (Fig. 8A). To rule out that the observed maturation defect in *Nr2f6*-deficient mice was related to the reduced number of metastasis, we pre-treated *Nr2f6*-deficient mice with IL-15C one day prior to the injection of B16-F10-*B2m*^*−/−*^ cells and administered IL-15C again on days 2 and 5 post-injection. After 16 days, metastasis formation was significantly reduced in IL-15C-treated *Nr2f6*-deficient mice compared to *Nr2f6*-deficient untreated mice, with 8 out of 10 treated mice showing no metastatic foci at all (Fig. 8A).

Tumor-infiltrating NK cell frequencies were similar between genotypes, as were frequencies in the blood and spleens of *Nr2f6*-deficient mice (Fig. 8B). NKp46 levels were consistently enhanced within *Nr2f6*-deficient NK cells in the tumor, blood and spleen (Fig. 8C). Of importance, whereas frequencies of terminal mature CD27^-^CD11b^+^KLRG1^+^ NK cells in the spleen were still reduced in *Nr2f6*-deficient mice, they were rescued to wild-type controls in the blood and the tumor (Fig. 8D).

Hence, *Nr2f6*-deficient NK cells exhibit superior functionality compared to their wild-type counterparts in the context of MHC-I deficient lung metastasis rejection.

## Discussion

NK cells have emerged as pivotal players in the development of immuno-oncologic treatments. Despite their potential, the full extent of their immunotherapeutic capability has not yet been fully established in the clinic [5, 6, 42–44]. In parallel to transcription factors that have been shown recently to regulate antitumor NK cell responses, such as HIF-1α, BACH2, or Zhx2, germline *Nr2f6*-deficient mice can reject B16-F10-*B2m*^*-/-*^ lung metastasis [43, 45–49]. Mechanistically, we identified NR2F6 as a negative regulator of *Ncr1* gene expression. In the absence of NR2F6, bone marrow-derived, peripheral, tumor-infiltrating, and in vitro expanded NK cells express significantly enhanced levels of the activating receptor NKp46. Along this line, blockage of NKp46 leads to the inhibition of NK cell-mediated killing of diverse cancer targets, and in the absence of *Ncr1*, mice displayed impaired tumor immunosurveillance [15, 50, 51]. In a clinical setting, tumor patients with elevated levels of NKp46 expression demonstrate a significantly improved prognosis compared to their counterparts characterized by low NKp46 levels [52]. Enhanced NK cell-specific NKp46 expression has also been observed in patients diagnosed with the autoimmune disease systemic lupus erythematosus (SLE) [53]. Our previous research has shown that deletion of NR2F6 in mice leads to a SLE-like immunopathological condition, especially in aged animals or following diverse immunization protocols [27, 31]. These findings raise the question of whether the augmented expression of NKp46 on NK cells, in addition to the primarily investigated CD4 follicular T helper and Th17 cell responses, might play a role in the progression of autoimmune diseases such as SLE, in germline *Nr2f6*-deficient mice.

A comprehensive understanding of how NRs regulate antitumor NK cell-specific responses has only recently begun. Retinoic acid and its receptor (RARα, NR1B1) regulate Granzyme B and NKp46 expression in the human NK-92 cell line [54, 55]. In the context of mouse tumor models, NR4A1 (Nuclear Receptor 4A1) has emerged as a significant player, mediating NK-cell dysfunction in hepatocellular carcinoma through the IFNγ/p-STAT1/IRF1 pathway [56]. Conversely, ROR (RAR-related orphan receptor) α has been identified as an essential factor for the maintenance and antitumor immunity of liver-resident NK cells [57].

In *Nr2f6*-deficient mice, the numbers of cDC1s and splenic macrophages are reduced. This results in a reduced IL-15 *trans*-presentation responsible for the maturation defect in NK cells observed under steady-state conditions. NK cell development and maturation in the bone marrow is independent of IL-15 *trans*-presentation by DCs or macrophages and, therefore, unaltered in *Nr2f6-*deficient mice [58]. As IL-15C treatment in vivo upregulates the expression of genes known to promote antitumor NK cell responses such as *Perf1*, *Stat5a*, Zeb2, *Fcgr3*, and *Itga2a* [59–63], while downregulating checkpoint inhibitors like *Lag3* and *Tigit* [64], it is plausible that *Nr2f6*-deficient NK cells exhibit enhanced efficacy during NK cell-mediated tumor immunosurveillance, due to their intrinsic elevation in *Ncr1* expression.

Considering that loss of NR2F6 enhances antitumor responses in T cells, especially during immune-checkpoint inhibition [29, 30], loss of NR2F6 in NK cells may be significant for tumors that lack potent T cell epitopes or have downregulated MHC-I expression as an escape mechanism, offering a potential avenue for a combinatorial therapeutic intervention.

## Material and methods

### Mice

*Nr2f6-*deficient mice on the C57BL/6 background have been described previously [29, 31, 32],. Mice were age (8-12 weeks) and sex-matched for individual experiments in a non-randomized manner. Animal procedures were approved by the Austrian Federal Ministry of Education, Science and Research (BMWFW-66.011/0064-WF/V/3b/2016; BMWFW-66.011/0112-WF/V/3b/2017, GZ: 2023-0.315.075, GZ: 2021-0.406.862, GZ: 2023-0.623.434, and GZ: 2024-0.885.804).

**B16-F10-*B2m***^***−/−***^ **melanoma lung metastasis** was performed as described previously [29].

### Tissue sampling

Spleens were homogenized through a 100 μm cell strainer in PBS (Sigma Aldrich-Aldrich, P5493-1L) supplemented with 3% FBS, 1% Penicillin/Streptomycin (Sigma-Aldrich, A2213). Blood was collected via the femoral artery during sacrifice or through the mandibular vein. Bone marrow (BM) was extracted from the femur and tibia via centrifugation (3000 g, 30 sec). If necessary, red blood cell lysis was performed using erythrocyte lysis buffer, as described previously [31, 32].

### Flow cytometry

Flow cytometric staining and analysis were performed as described previously [31, 32]. The complete list of used antibodies can be found under supplementary methods Table 1.

### NK Isolation and in vitro culture

According to the manufacturer’s instructions, NK cells were negatively isolated from total splenocytes using the mouse NK cell isolation kit (Miltenyi Biotec, 130 115 818). 1–2 × 10^5^ NK cells were cultured in 96-well plates in IMDM (Sigma-Aldrich, I3390) supplemented with 10% FBS, 1x non-essential amino-acids, and 50 µM β-mercaptoethanol (Sigma-Aldrich, M3148-25ML) in the presence of 50 ng/ml IL-15 (Biolegend, 566302), or 50 ng/ml IL-15 and 25 ng/ml IL-18 (BioLegend, 767002*)*, or 25 ng/ml IL-2 (BioLegend, 575402).

### NK cell stimulation

Total splenocytes or isolated NK cells were stimulated for 5 hours under the following conditions: 25 ng/ml IL-12 (BioLegend, 577002) and 25 ng/ml IL-18 (BioLegend, 767002), 5 µg/ml αNKp46, or co-cultured with 1×10^4^ B16 cells for 24 h. For intracellular cytokine measurement, GolgiPlug™ was added 4 h before the harvest.

### ChIP

Chromatin immunoprecipitation assays were performed as previously described [31].

### In vivo NK cell activation and IFN-γ secretion

To activate NK cells in vivo, age-matched mice were injected i.v. with 2 μg LPS (LPS-SM Ultrapure (LPS from S. minnesota R595), InvivoGen). Mice were euthanized 2.5 hr before the 5 hr time points, to culture RBC-lysed splenocytes in the presence of brefeldin A (BFA; 10 μg/ml; Sigma-Aldrich) for the remaining time. This allowed measuring direct IFN-γ release by NK cells in the absence of artificial restimulation [2, 65].

### IL-15 complex treatment

Wild-type or *Nr2f6*-deficient mice were i.v. injected with 2 µg IL-15 complex (2:9 ratio: 0,36 µg recombinant mIL-15 (BioLegend, 566302) and 1,63 µg recombinant mouse IL-15R alpha (R&D Systems, 551-MR-100) complexed for 30 minutes at 37°C) or PBS as described previously every third day and analyzed after 9 days [39]. During tumor formation IL-15C was injected on day -1 and then on day 2 and 5 after B16-F10-*B2m*^*-/-*^ melanoma lung metastasis induction.

### Cell sorting and RNA preparation

Total splenic NK cells were sorted as live/Lin^-^/CD45^+^/NK1.1^+^/NKp46^+^/CD49b^+^ on a FACS Aria III (BD Biosciences). RNA from sorted cells was extracted using the RNeasy mini Kit (Qiagen, 74104). RNA quantity and integrity were determined by Agilent 5400, and only samples with > 100 ng RNA and RNA integrity number > 4 were used. PolyA enrichment, non-directional library preparation, and Illumina sequencing were performed by Novogene.

Gene counts were derived based on the number of reads mapped to each gene using featureCounts (v1.5.0-p3) [66]. Differentially expression (DE) analysis was performed using the DESeq2 R package (1.20.0) [67]. The resulting *p* values were adjusted using the Benjamini and Hochberg’s approach to control the false discovery rate. Genes with an adjusted *p* value <=0.05 were considered as differentially expressed [68]. DESeq2 normalized gene expression values were used to perform KEGG WebGestalt [69] and GSEA [70, 71].

### Statistical analysis

Statistical analysis of experimental data was performed using Prism 10.2.3. The Gaussian distribution of the data was assessed using the Shapiro-Wilk test, and variance discrepancies were evaluated with the F-test. For gaussian distributed samples, differences were analyzed using an unpaired Student’s *t*-test, while non-gaussian distributed samples were compared using the Mann-Whitney *U* test. Randomization, blinding, or sample size estimation tests were not applied to our animal studies.

## Supplementary information

Supplementary Files

## Data Availability

The raw data supporting the conclusions of this article will be made available by the authors without undue reservation. RNAseq primary data deposition is found under GEO accession GSE263734.
