# Supplementary material for: Regulation of NK cell development, maturation, and antitumor responses by the nuclear receptor NR2F6
Source: Cell Death Dis. 2025 Feb 7;16(1):77. doi: 10.1038/s41419-025-07407-4 (PMC11806049; doi:10.1038/s41419-025-07407-4)
Supplement: Supplementary file 1 — Supplementary Files [file 41419_2025_7407_MOESM1_ESM.pdf]

Supplementary Files

Regulation of NK cell development, maturation, and anti-tumor responses by the nuclear receptor NR2F6

Johannes Woelk[1], Florian Hornsteiner[2], Stephanie Aschauer-Wallner[3], Patrizia Stoitzner[2], Gottfried Baier[1] and Natascha Hermann-Kleiter[1*,]

[1] Institute of Cell Genetics, Department for Genetics and Pharmacology, Medical University of Innsbruck, Innsbruck, Austria.

[2] Department of Dermatology, Venereology & Allergology, Medical University of Innsbruck, Innsbruck, Austria.

[3] Laboratory of Tumor Immunology, Tyrolean Cancer Institute & Internal Medicine V, Medical University of Innsbruck, 6020 Innsbruck, Austria. Current address: Regenerative Medicine, Spinal Cord Injury and Tissue Regeneration Center Salzburg, Paracelsus Medical University Salzburg, Salzburg, Austria.

***Corresponding author**: Natascha Hermann-Kleiter, email: natascha.kleiter@i-med.ac.at (N-HK)

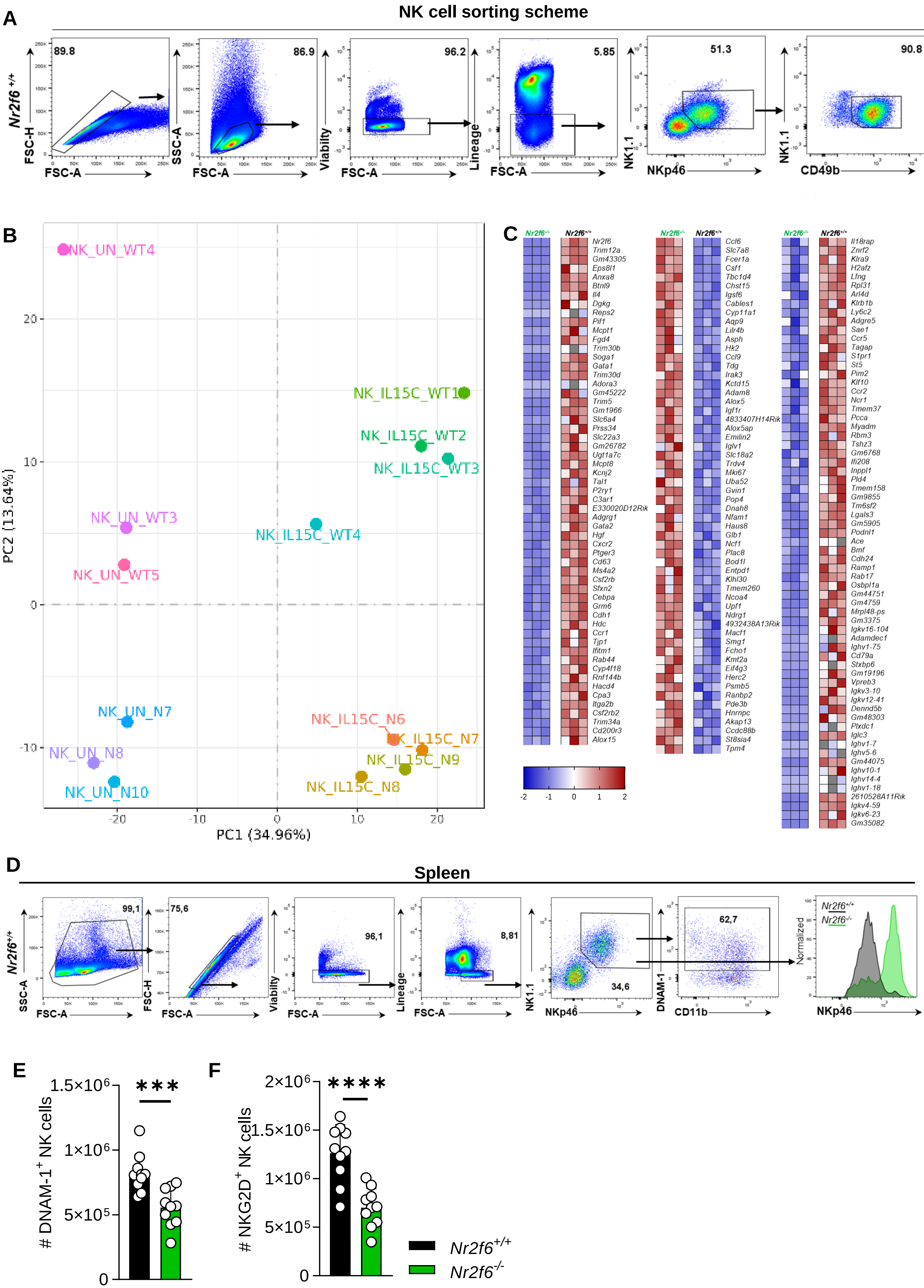

A
NK cell sorting scheme
Nr2f6 +/+
FSC-H
FSC-A
89.8
SSC-A
86.9
Viability
96.2
Lineage
5.85
NK1.1
NKp46
51.3
CD49b
90.8
B
PC1 (34.96%)
PC2 (13.64%)
NK_UN_WT4
NK_IL15C_WT1
NK_IL15C_WT2
NK_IL15C_WT3
NK_UN_WT3
NK_IL15C_WT4
NK_UN_WT5
NK_UN_N7
NK_IL15C_N6
NK_IL15C_N7
NK_UN_N8
NK_IL15C_N9
NK_UN_N10
NK_IL15C_N8
C
Nr2f6-/-
Nr2f6+/+
Nr2f6 Trim12a Gm43305 Eps8l1 Anxa8 Btnl9 Il4 Dgkg Reps2 Pif1 Mcpt1 Fgd4 Trim30b Soga1 Gata1 Trim30d Adora3 Gm45222 Trim5 Gm1966 Slc6a4 Prss34 Slc22a3 Gm26782 Ugt1a7c Mcpt8 Kcnj2 Tal1 P2ry1 C3ar1 E330020D12Rik Adgrg1 Gata2 Hgf Cxcr2 Ptger3 Cd63 Ms4a2 Csf2rb Sfxn2 Cebpa Grm6 Cdh1 Hdc Ccr1 Tjp1 Ifitm1 Rab44 Cyp4f18 Rnf144b Hacd4 Cpa3 Itga2b Csf2rb2 Trim34a Cd200r3 Alox15
Ccl6 Slc7a8 Fcer1a Csf1 Tbc1d4 Chst15 Igsf6 Cables1 Cyp11a1 Aqp9 Lilr4b Asph Hk2 Ccl9 Tdg Irak3 Kctd15 Adam8 Alox5 Igf1r 4833407H14Rik Alox5ap Emilin2 Iglv1 Slc18a2 Trdv4 Mki67 Uba52 Gvin1 Pop4 Dnah8 Nfam1 Haus8 Glb1 Ncf1 Plac8 Bod1l Entpd1 Klhl30 Tmem260 Ncoa4 Upf1 Ndrg1 4932438A13Rik Macf1 Smg1 Fcho1 Kmt2a Eif4g3 Herc2 Psmb5 Ranbp2 Pde3b Hnrnpc Akap13 Ccdc88b St8sia4 Tpm4
Il18rap Znrf2 Klra9 H2afz Lfng Rpl31 Arl4d Klrb1b Ly6c2 Adgre5 Sae1 Ccr5 Tagap S1pr1 St5 Pim2 Klf10 Ccr2 Ncr1 Tmem37 Pcca Myadm Rbm3 Tshz3 Gm6768 Ifi208 Inppl1 Pld4 Tmem158 Gm9855 Tm6sf2 Lgals3 Gm5905 Podnl1 Ace Bmf Cdh24 Ramp1 Rab17 Osbpl1a Gm44751 Gm4759 Mrpl48-ps Gm3375 Igkv16-104 Adamdec1 Ighv1-75 Cd79a Stxbp6 Gm19196 Vpreb3 Igkv3-10 Igkv12-41 Dennd5b Gm48303 Plxdc1 Iglc3 Ighv1-7 Ighv5-6 Gm44075 Ighv10-1 Ighv14-4 Ighv1-18 2610528A11Rik Igkv4-59 Igkv6-23 Gm35082
-2 -1 0 1 2
D
Spleen
Nr2f6+/+
SSC-A
FSC-A
99,1
FSC-H
75,6
Viability
96,1
Lineage
8,81
NK1.1
NKp46
34,6
DNAM-1
CD11b
62,7
Normalized
Nr2f6+/+
Nr2f6-/-
NKp46
E
DNAM-1+ NK cells
1.5×10⁶
1×10⁶
5×10⁵
0
***
F
NKG2D+ NK cells
2×10⁶
1.5×10⁶
1×10⁶
5×10⁵
0
****
Nr2f6+/+
Nr2f6-/-

Supplementary Figure 1: **Characterization of *Nr2f6*-deficient NK cells via RNA-Seq analysis.**

(A) Gating strategy for splenic NK cell fluorescence-activated cell sorting from wild-type (*Nr2f6*$^{+/+}$) or *Nr2f6*-deficient (*Nr2f6*$^{-/-}$) mice. Single events were selected, debris was excluded, and only viable cells were included from total splenocytes. Lin^{+} cells (CD3^{+}CD19^{+}) were excluded and NK cells were identified by NK1.1, NKp46 and CD49b expression.

(B) Principal component analysis (PCA) of sorted splenic NK cells **(A)** from healthy wild-type (*Nr2f6*$^{+/+}$) and *Nr2f6*-deficient (*Nr2f6*$^{-/-}$), or IL-15C treated wild-type (*Nr2f6*$^{+/+}$) and *Nr2f6*-deficient (*Nr2f6*$^{-/-}$) (UN: n = 3, IL-15C: n = 4) mice.

(C) Heatmap of differentially expressed genes (DEG) of wild-type (*Nr2f6*$^{+/+}$) or *Nr2f6*-deficient (*Nr2f6*$^{-/-}$) splenic NK cells. All genes were z-score normalized, and DEGs were defined by DESeq2 (adjusted p-value (padj) < 0.05) (n = 3 per genotype).

(D) Gating strategy for splenic and blood NK cells from wild-type (*Nr2f6*$^{+/+}$) or *Nr2f6*-deficient (*Nr2f6*$^{-/-}$) mice. Debris was excluded, single events were selected and only viable cells were included from total splenocytes or blood lymphocytes. Lin^{+} cells (CD3^{+}CD19^{+}) were excluded and NK cells were identified by expression of NK1.1 and NKp46. NK cells were further characterized by e.g., expression of NKp46, DNAM-1 or NKG2D.

(E) Quantification of total cell counts of DNAM-1^{+} splenic NK cells (CD3^{-}CD19^{-}NK1.1^{+}NKp46^{+}) in wild-type (*Nr2f6*$^{+/+}$) or *Nr2f6*-deficient (*Nr2f6*$^{-/-}$) mice.

(F) Quantification of total cell counts of NKG2D^{+} splenic NK cells (CD3^{-}CD19^{-}NK1.1^{+}NKp46^{+}) in wild-type (*Nr2f6*$^{+/+}$) or *Nr2f6*-deficient (*Nr2f6*$^{-/-}$) mice.

(A-C) RNA sequencing and all downstream analyses were performed on splenic NK cells from n=3 per genotype. (E, F) Representative data is shown as pooled experiments of at least three independent experiments n =10. Each dot represents the data of an individual mouse. Results are shown as mean ± SD. The normality of data was evaluated by the Shapiro-Wilk test. An asterisk indicates statistically significant differences between genotypes calculated using Student's *t*-test. A p-value < 0.05 was considered statistically significant. ***p < 0.001 ****p < 0.0001.

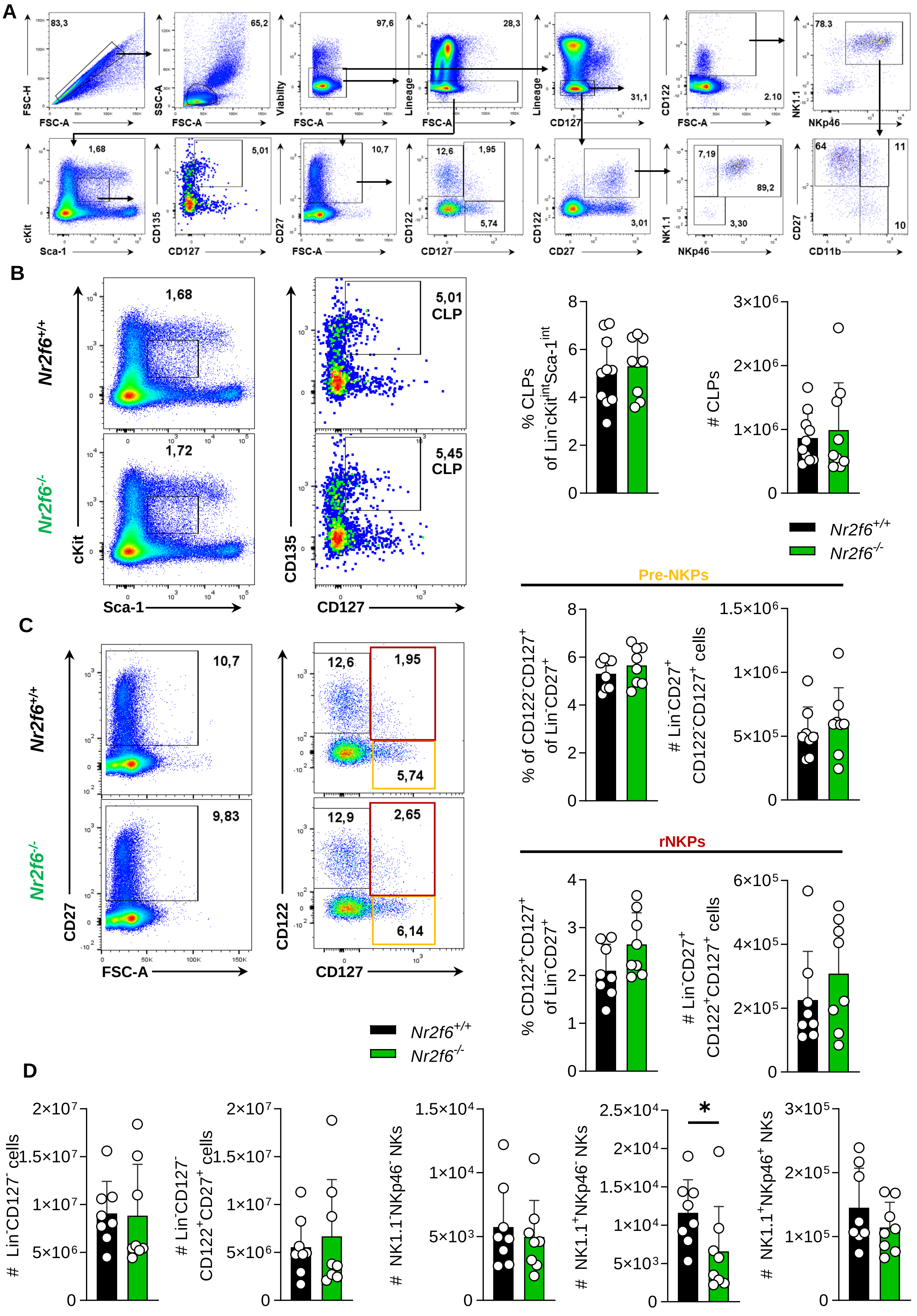
A
B
C
D
Nr2f6+/+
Nr2f6-/-
CLP
Pre-NKPs
rNKPs
% CLPs of Lin-cKitintSca-1int
# CLPs
% of CD122-CD127+ of Lin-CD27+
# Lin-CD27+ CD122-CD127+ cells
% CD122+CD127+ of Lin-CD27+
# Lin-CD27+ CD122+CD127+ cells
# Lin-CD127- cells
# Lin-CD127- CD122+CD27+ cells
# NK1.1-NKp46- NKs
# NK1.1+NKp46- NKs
# NK1.1+NKp46+ NKs
*

Supplementary Figure 2: ***Nr2f6*-deficient mice have normal NK cell development in the bone marrow.**

(A) Gating strategy from singlet, cells, live, lineage$^-$, CLP (Lin$^-$CD117^{int}Sca1^{int}CD135$^+$CD127$^+$), different NK progenitor (Lin$^-$CD27$^+$CD122$^-$CD127$^+$; Lin$^-$CD27$^+$CD122$^+$CD127$^+$), immature (Lin$^-$CD127$^-$CD122$^+$CD27$^+$NK1.1$^-$NKp46$^-$; Lin$^-$CD127$^-$CD122$^+$CD27$^+$NK1.1$^+$NKp46$^-$) and mature NK (Lin$^-$CD127$^-$CD122$^+$NK1.1$^+$NKp46$^+$CD27$^+$CD11b$^-$; Lin$^-$CD127$^-$CD122$^+$NK1.1$^+$NKp46$^+$CD27$^+$CD11b$^+$; Lin$^-$CD127$^-$CD122$^+$NK1.1$^+$NKp46$^+$CD27$^-$CD11b$^+$) populations in the bone marrow from femur and tibia of healthy wild-type (*Nr2f6*$^{+/+}$) or *Nr2f6*-deficient (*Nr2f6*$^{-/-}$) mice.

(B) Representative dot plots, quantification of frequency of parent and total cell counts of CLP (CD135$^+$CD127$^+$) of wild-type (*Nr2f6*$^{+/+}$) or *Nr2f6*-deficient (*Nr2f6*$^{-/-}$) bone marrow-derived Lin$^-$CD117^{int}Sca1^{int} cells.

(C) Representative dot plots, quantification of the frequency of parent and total cell counts of CD122$^-$CD127$^+$ pre-NK progenitors and CD122$^+$CD127$^+$ refined NK progenitors (rNKPs) out of wild-type (*Nr2f6*$^{+/+}$) *or Nr2f6*-deficient (*Nr2f6*$^{-/-}$) bone marrow-derived Lin$^-$CD27$^+$ cells.

(D) Quantification of total cell counts of Lin$^-$CD127$^-$ cells, Lin$^-$CD127$^-$CD122$^+$CD27$^+$ NK-progenitors, Lin$^-$CD127$^-$CD122$^+$CD27$^+$ stage A-C NK cells (NK1.1$^-$NKp46$^-$, NK1.1$^+$NKp46$^-$, NK1.1$^+$NKp46$^+$) of wild-type (*Nr2f6*$^{+/+}$) or *Nr2f6*-deficient (*Nr2f6*$^{-/-}$) bone marrow derived cells.

(B) Representative data is shown as pooled experiments of at least three independent experiments n =10 (*Nr2f6*$^{+/+}$) and n=9 (*Nr2f6*$^{-/-}$)

(C, D) Representative data is shown as pooled experiments of at least three independent experiments n = 8. Each dot represents the data of an individual mouse. Results are shown as mean ± SD. An asterisk indicates statistically significant differences between genotypes calculated using Student's t-test or Mann-Whitney *U* test. A p-value < 0.05 was considered statistically significant. *p < 0.05

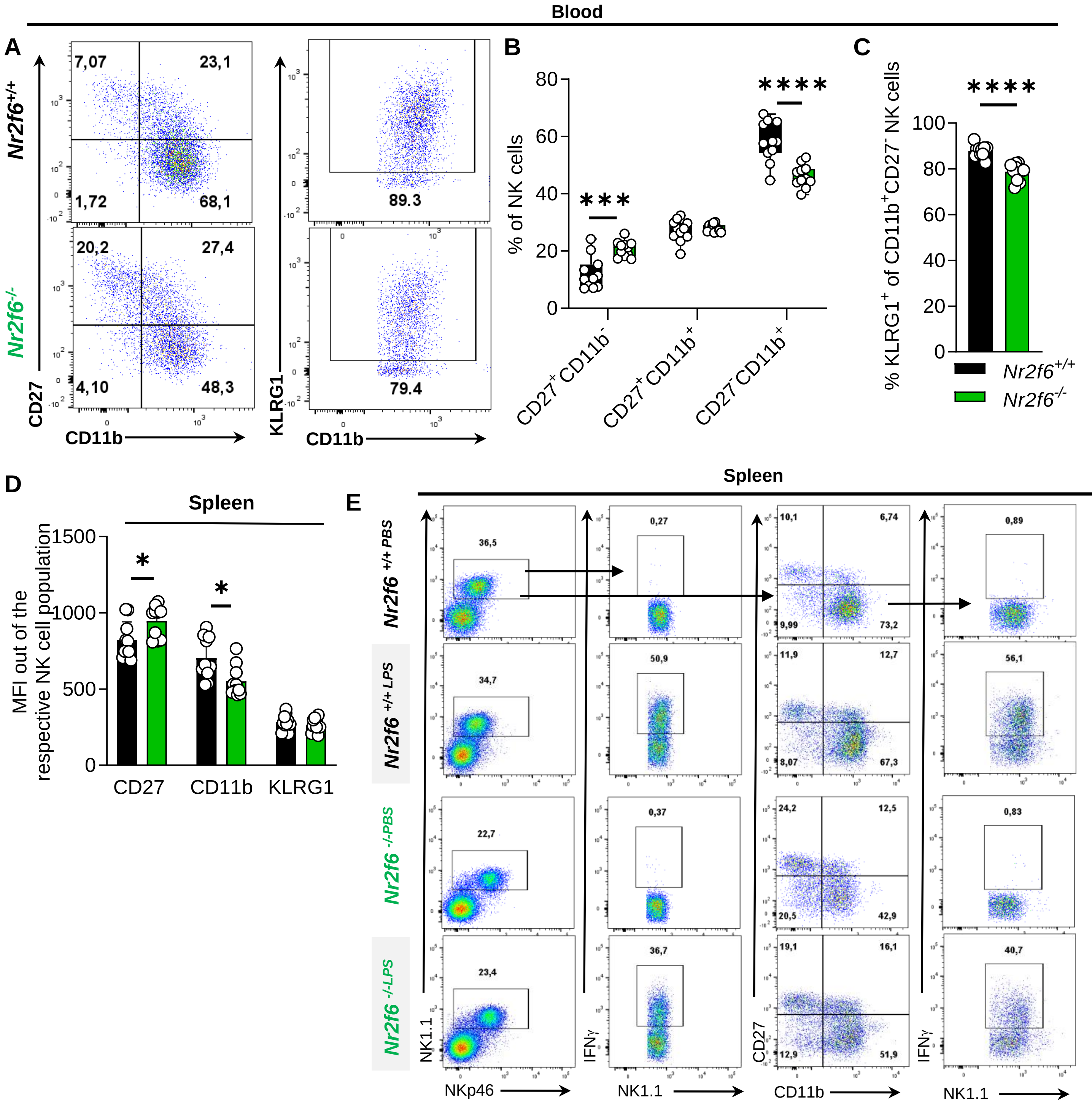
Blood
A
Nr2f6+/+
7,07
23,1
1,72
68,1
89.3
Nr2f6-/-
20,2
27,4
4,10
48,3
79.4
CD27
CD11b
KLRG1
CD11b
B
% of NK cells
***
****
CD27+CD11b-
CD27+CD11b+
CD27-CD11b+
C
% KLRG1+ of CD11b+CD27- NK cells
****
Nr2f6+/+
Nr2f6-/-
D
Spleen
MFI out of the respective NK cell population
*
*
CD27
CD11b
KLRG1
Spleen
E
Nr2f6 +/+ PBS
Nr2f6 +/+ LPS
Nr2f6 -/-PBS
Nr2f6 -/-LPS
36,5
0,27
10,1
6,74
9,99
73,2
0,89
34,7
50,9
11,9
12,7
8,07
67,3
56,1
22,7
0,37
24,2
12,5
20,5
42,9
0,83
23,4
36,7
19,1
16,1
12,9
51,9
40,7
NK1.1
NKp46
IFNγ
NK1.1
CD27
CD11b
IFNγ
NK1.1

Supplementary Figure 3: **Terminal NK cell maturation block in the blood of *Nr2f6*-deficient mice.**

(A) Representative dot-plots of wild-type (*Nr2f6*$^{+/+}$) or *Nr2f6*-deficient (*Nr2f6*$^{-/-}$) NK1.1^{+}NKp46^{+}, CD27^{-}CD11b^{-}, CD27^{+}CD11b^{-}, CD27^{+}CD11b^{+}, mature CD27^{-}CD11b^{+} and terminal mature CD11b^{+}KLRG1^{+} NK cell populations in the blood.

(B) Quantification of wild-type (*Nr2f6*$^{+/+}$) or *Nr2f6*-deficient (*Nr2f6*$^{-/-}$) NK1.1^{+}NKp46^{+}, CD27^{-}CD11b^{-}, CD27^{+}CD11b^{-}, CD27^{+}CD11b^{+}, mature CD27^{-}CD11b^{+} and **(C)** terminal mature CD11b^{+}KLRG1^{+} blood derived NK cell frequencies.

(D) Quantification of the MFI of CD27, CD11b and KLRG1 in wild-type (*Nr2f6*$^{+/+}$) or *Nr2f6*-deficient (*Nr2f6*$^{-/-}$) splenic NK cells (CD3^{-}CD19^{-}NK1.1^{+}NKp46^{+}).

(E) Representative dot-plots of splenic NK cells (CD45^{+}CD3^{-}NK1.1^{+}NKp46^{+}) and IFNγ-producing NK cells in wild-type (*Nr2f6*$^{+/+}$) or *Nr2f6*-deficient (*Nr2f6*$^{-/-}$) mice after PBS or LPS injection.

(B, C) Representative data is shown as pooled experiments of at least two independent experiments $n = 11$. (D) Representative data is shown as pooled experiments of at least two independent experiments $n = 10$. Each dot represents the data of an individual mouse. Results are shown as mean ± SD. An asterisk indicates statistically significant differences between genotypes calculated using Student's t test. A p-value < 0.05 was considered statistically significant. ***p < 0.001; ****$p < 0.0001$.

A

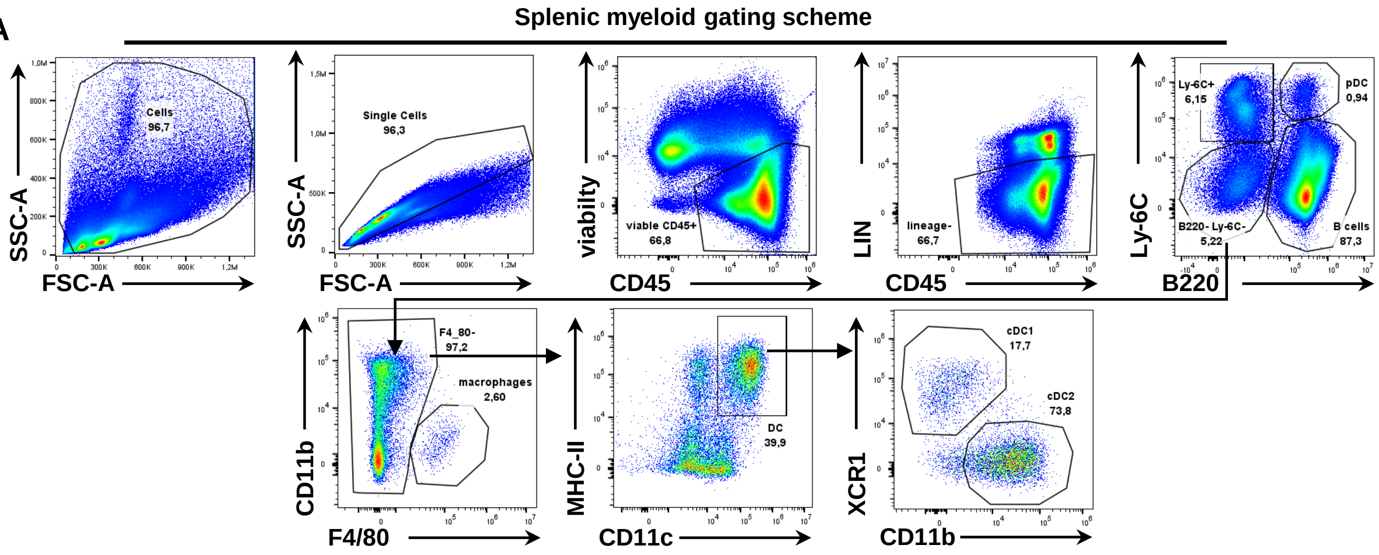

Supplementary Figure 4:

(A) Gating strategy of splenic derived myeloid cell populations. Single events were selected, debris was excluded, and only viable cells were included from total splenocytes.

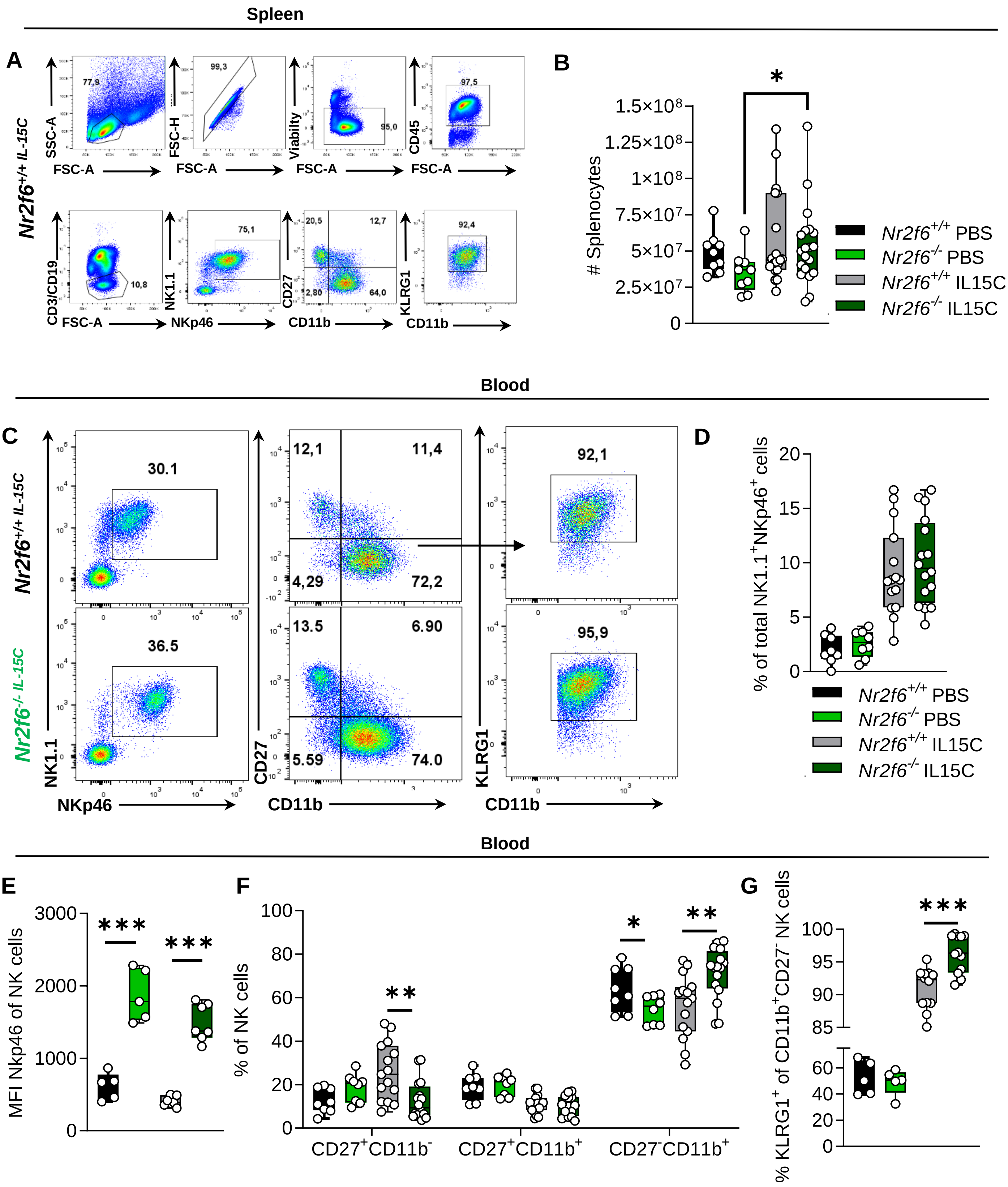

Spleen
A
Nr2f6+/+ IL-15C
SSC-A
FSC-A
77,9
FSC-H
99,3
Viability
95,0
CD45
97,5
CD3/CD19
10,8
NK1.1
NKp46
75,1
CD27
CD11b
20,5
12,7
2,80
64,0
KLRG1
92,4
B
*
Splenocytes
1.5×10^8
1.25×10^8
1×10^8
7.5×10^7
5×10^7
2.5×10^7
0
Nr2f6+/+ PBS
Nr2f6-/- PBS
Nr2f6+/+ IL15C
Nr2f6-/- IL15C
Blood
C
Nr2f6+/+ IL-15C
Nr2f6-/- IL-15C
30.1
12,1
11,4
4,29
72,2
92,1
36.5
13.5
6.90
5.59
74.0
95,9
NK1.1
NKp46
CD27
CD11b
KLRG1
CD11b
D
% of total NK1.1+NKp46+ cells
20
15
10
5
0
Nr2f6+/+ PBS
Nr2f6-/- PBS
Nr2f6+/+ IL15C
Nr2f6-/- IL15C
Blood
E
MFI Nkp46 of NK cells
3000
2000
1000
0
***
***
F
% of NK cells
100
80
60
40
20
0
**
*
**
CD27+CD11b-
CD27+CD11b+
CD27-CD11b+
G
% KLRG1+ of CD11b+CD27- NK cells
100
95
90
85
60
40
20
0
***

Supplementary Figure 5: IL-15C treatment of *Nr2f6*-deficient mice rescues NK maturation and effector responses

(A) Gating strategy of NK cell maturation following IL-15C treatment in the spleen or blood of wild-type (*Nr2f6*$^{+/+}$) or *Nr2f6*-deficient (*Nr2f6*$^{-/-}$) mice.

(B) Quantification of wild-type (*Nr2f6*$^{+/+}$) or *Nr2f6*-deficient (*Nr2f6*$^{-/-}$) splenocyte numbers after PBS or 3 x IL-15/IL-15Rα-complex treatment on day 9.

(C) Representative dot-plots of wild-type (*Nr2f6*$^{+/+}$) or *Nr2f6*-deficient (*Nr2f6*$^{-/-}$) NK1.1^{+}NKp46^{+}, CD27^{-}CD11b^{-}, CD27^{+}CD11b^{-}, CD27^{+}CD11b^{+}, mature CD27^{-}CD11b^{+} and terminal mature CD11b^{+}KLRG1^{+} NK cell populations in the blood following 3 x IL-15/IL-15Rα-complex or PBS treatment (out of CD45^{+}CD3^{-}CD19^{-}) on day 9.

(D) Quantification of wild-type (*Nr2f6*$^{+/+}$) or *Nr2f6*-deficient (*Nr2f6*$^{-/-}$) NK1.1^{+}NKp46^{+}, blood derived NK cell frequencies following 3 x IL-15/IL-15Rα-complex or PBS treatment on day 9.

(E) Quantification of the MFI of NKp46 out of wild-type (*Nr2f6*$^{+/+}$) or *Nr2f6*-deficient (*Nr2f6*$^{-/-}$) NK1.1^{+}NKp46^{+} NK cells in the blood either PBS or IL-15C treated.

(F) Quantification of blood derived CD27^{+}CD11b^{-}, CD27^{+}CD11b^{+}, mature CD27^{-}CD11b^{+} and **(G)** terminal mature CD11b^{+}KLRG1^{+} NK cell frequencies of wild-type (*Nr2f6*$^{+/+}$) or *Nr2f6*-deficient (*Nr2f6*$^{-/-}$) following 3 x IL-15/IL-15Rα-complex or PBS treatment (out of CD45^{+}CD3^{-}CD19^{-}) on day 9.

(B) Representative data are shown as pooled experiments of five independent experiments *n* = 9 (PBS treated wild-type (*Nr2f6*$^{+/+}$) or *Nr2f6*-deficient (*Nr2f6*$^{-/-}$) mice) and *n*=19 (IL15C treated wild-type (*Nr2f6*$^{+/+}$) mice) or *n*=20 (IL-15C treated *Nr2f6*-deficient (*Nr2f6*$^{-/-}$) mice). (D, F, G) Representative data are shown as pooled experiments of four independent experiments *n* = 8 (PBS treated wild-type (*Nr2f6*$^{+/+}$) or *Nr2f6*-deficient (*Nr2f6*$^{-/-}$) mice) and *n*=15 (IL15C treated wild-type (*Nr2f6*$^{+/+}$) mice) or *n*=16 (IL-15C treated *Nr2f6*-deficient (*Nr2f6*$^{-/-}$) mice). (E) The representative data shown are from two pooled experiments out of five replicative experiments, with n =2-7 per group and experiment. *n* = 5 (PBS treated wild-type (*Nr2f6*$^{+/+}$) or *Nr2f6*-deficient (*Nr2f6*$^{-/-}$) mice) and *n*=7 (IL15C treated wild-type (*Nr2f6*$^{+/+}$) or *Nr2f6*-deficient (*Nr2f6*$^{-/-}$) mice). Each dot represents the data of an individual mouse. Results are shown as mean ± SD. An asterisk indicates statistically significant differences between genotypes calculated using Student's t-test, or Mann-Whitney *U* test. A p-value < 0.05 was considered statistically significant. *p < 0.05; **p < 0.01; ***p < 0.001.

A

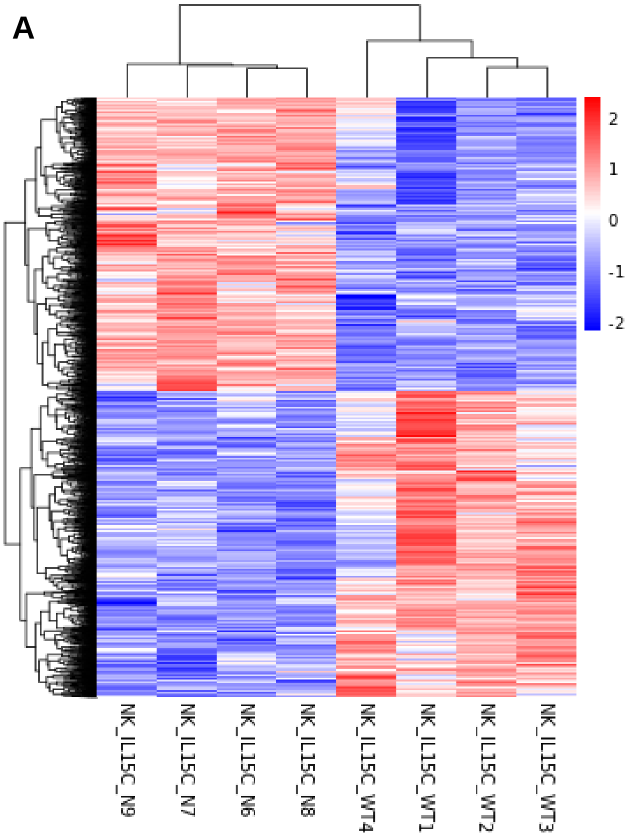

Supplementary Figure 6: Characterization of *Nr2f6*-deficient NK cells following IL-15C treatment via RNA-Seq analysis.

(A) Heatmap of differentially expressed genes (DEG) in splenic NK cells from IL-15C treated wild-type (*Nr2f6*$^{+/+}$) or *Nr2f6*-deficient (*Nr2f6*$^{-/-}$) mice. All genes were z-score normalized, and DEGs were defined by DESeq2 (adjusted p-value (padj) < 0.05) (n = 4 per genotype).

Table 1: Antibody list for flow cytometry and sorting

AB	Fluorochrome	Clone	Company	Order Number
B220	Biotin	RA3-6B2	BioLegend	103204
CCR5	PE	HM-CCR5	BioLegend	107005
CD117	APC-Cy7	2B8	BioLegend	105825
CD11b	BV510	M1/70	BioLegend	101245
CD11b	PerCP-Cy5.5	M1/70	BioLegend	101229
CD11b	BV605	M1/70	BioLegend	101257
CD11c	Biotin	N418	BioLegend	117304
CD11c	BV510	N418	BioLegend	117353
CD122	APC-Cy7	TM-b1	BioLegend	123221
CD127 (IL-7Ra)	PE	SB/199	BioLegend	121111
CD127 (IL-7Ra)	PE-Cy7	A7R34	BioLegend	135014
CD135 (Flk2, Flt3)	APC	A2F10	BioLegend	135310
CD19	Biotin	6D5	BioLegend	115503
CD19	Pacific Blue	1D3	BioLegend	115523
CD19	FITC	1D3	BioLegend	152403
CD226 (DNAM-1)	PE-Cy7	10E5	BioLegend	128811
CD27	Pacific Blue	LG.3A10	BioLegend	124217
CD27	FITC	LG3AC	eBiosciences	11-0271-82
CD3	Pacific Blue	17A2	BioLegend	100214
CD3	PerCP-Cy5.5	2C11	BioLegend	100327
CD3e	Biotin	145-2C11	BioLegend	100304
CD45	V500	30-F11	BD	561487
CD45	AF700	30-F11	BioLegend	103127
CD49b	PB	DX5	BD	563063
F4/80	PE/Dazzle 594	BM8	BioLegend	123145
GR1 (Ly6G Ly6C)	Biotin	RB6-8C5	BioLegend	108404
IL15 RA	AF647/APC	6B4C88	BioLegend	153505
KLRG1	PE-Cy7	2F1/KLRG1	BioLegend	138415
Langerin	PE	4C7	BioLegend	144203
Ly-6C	BV421	HK1.4	BioLegend	128032
MHC-II (I-A/I-E)	PE-Cy7	M5/114	BioLegend	107629
NK1.1	APC	PK136	BioLegend	108709
NK1.1	PerCP	PK136	BioLegend	108725
NK1.1	PerCP-Cy5.5	PK136	BioLegend	108727
Nkp46 (CD335)	APC	29A1.4	BioLegend	137607
Nkp46 (CD335)	BV605	29A1.5	BioLegend	137619
Nkp46 (CD335)	PE	29A1.4	BioLegend	137603
Sca-1	PE	D7	BioLegend	108107
Ter119	Biotin	Ter-119	BioLegend	116204
XCR1	BV650	ZET	BioLegend	148220
Fixable Viability Stain	575V		BD	565694
Fixable Viability Stain	780		BD	565388
Granzyme B	PE	NGZB	eBioscience	12-8898-80
IFNγ	PE-Cy7	XMG1.2	Biolegend	505825
Ki-67	PE	16A8	Biolegend	652404
Perforin	APC	S16009A	Biolegend	154303
TNFα	BV510	MP6-XT22	Biolegend	560659

Supplementary Figure 7: Table 1: Antibody list for flow cytometry and sorting
